# The bovine ocular microbiome: a multi-approach study of composition and antimicrobial activity

**DOI:** 10.1186/s42523-026-00587-0

**Published:** 2026-06-15

**Authors:** Samat Amat, Devin B. Holman, Sarah M. Luecke, Katherine E. Gzyl, Muhammad Anas, Gerald Stokka

**Affiliations:** 1 Department of Microbiological Sciences, North Dakota State University, Fargo, ND 58108 USA; 2 Lacombe Research and Development Centre, Agriculture and Agri-Food Canada, Lacombe, AB Canada; 3 Department of Animal Sciences, North Dakota State University, Fargo, ND 58102 USA

**Keywords:** Pinkeye, Ocular microbiome, Infectious bovine keratoconjunctivitis, Metagenomic sequencing, Whole-genome sequencing, Antimicrobial resistance, Cattle

## Abstract

**Background:**

Despite widespread use of antimicrobials and vaccines, the incidence of infectious bovine keratoconjunctivitis (IBK), or pinkeye, continues to increase in North American beef cow-calf operations. Recent research suggests that there is potential for the commensal ocular microbiome to help mitigate IBK. Therefore, this study characterized the ocular microbiome of cattle with and without IBK using culture-based methods and shotgun metagenomic sequencing and assessed the ability of commensal bacteria to inhibit *Moraxella* spp. in vitro. Ocular swabs (*n* = 143) were collected from IBK-affected (*n* = 102) and healthy cattle (*n* = 41) before antimicrobial treatment from North Dakota herds. Bacteria were cultured aerobically and anaerobically on five different media and the isolates were identified. A subset of swabs (37 IBK-affected; 12 healthy) underwent shotgun metagenomic sequencing. The genomes of 31 isolates, including *Moraxella bovoculi*, *Moraxella bovis*, and commensal bacteria, were also sequenced. Fifty-two commensal isolates were screened for inhibition of *Moraxella* spp. using an agar slab method, with five isolates further tested by qPCR for inhibition in the presence of the culturable ocular microbiome.

**Results:**

The 351 bacterial isolates taxonomically identified represented 61 genera from three phyla. The majority of isolates belonged to *Bacillus* (25.9%), *Streptococcus* (11.1%), *Staphylococcus* (10.1%), and *Moraxella* (9.4%) genera. Shotgun metagenomic analysis revealed significant differences in ocular microbial species composition between IBK-affected and healthy cattle (R² = 0.05; *P* = 0.015) based on Bray-Curtis dissimilarity. Dominant bacterial species included *Cutibacterium acnes*, *Mannheimia pernigra*, *Mesomycoplasma bovoculi*, *Moraxella bovis*, and *Moraxella bovoculi*. Eight bacterial species, including *Bifidobacterium globosum* and *Bacillus licheniformis*, were more abundant in healthy cattle, while *Arthrobacter luteus* was enriched in IBK cases. Thirty-seven high-quality metagenome-assembled genomes were also recovered, with 27% classified as *Mesomycoplasma bovoculi*. *Moraxella* spp. genomes exhibited strain-specific antimicrobial resistance and virulence gene diversity. Seventeen commensal isolates inhibited *Moraxella*, with *Weizmannia coagulans*, *Lentilactobacillus buchneri*, and *Paenibacillus polymyxa* showing strong activity. Selected isolates maintained inhibitory effects in co-culture with the ocular microbiome.

**Conclusion:**

The ocular surface of beef cattle is inhabited by a diverse microbiome that includes several bacterial strains that have the potential to be used as therapeutics to inhibit IBK pathogens.

**Supplementary Information:**

The online version contains supplementary material available at 10.1186/s42523-026-00587-0.

## Introduction

Pinkeye, clinically known as infectious bovine keratoconjunctivitis (IBK), is a highly contagious eye disease that can cause inflammation of the cornea and conjunctiva, negatively affecting animal welfare and reducing profitability in the global beef cattle industry [1–3]. Bacterial pathogens, together with environmental factors (e.g., season, ultraviolet radiation, dust, and flies) and host factors (e.g., age, genetics, and immune status), influence the pathogenesis of IBK in cattle [4–6]. *Moraxella bovis* is considered the primary bacterial agent involved in the development of IBK [7–9]. This species can spread rapidly within cattle herds through direct contact or environmental transmission [10].

*Moraxella bovoculi* is also an important IBK pathogen [11, 12]. As such, *Moraxella bovis* and *Moraxella bovoculi* have been the main targets for vaccine and antimicrobial-based IBK prevention and control in beef cattle. Currently available vaccines used to prevent IBK outbreaks in cattle herds have shown limited effectiveness in controlled trials [13, 14]. Therefore, antimicrobials have been the primary means of preventing and controlling the spread of these *Moraxella* spp. within a cattle herd. However, a recent report showing multidrug resistance in *Moraxella bovis* and *Moraxella bovoculi* strains from the bovine ocular surface [15] highlights the necessity of developing antimicrobial alternatives to control IBK [16].

Emerging evidence from culture-independent, high-throughput sequencing studies in humans suggests that the eye harbors a relatively diverse and dynamic microbial community, and that this ocular microbiota may be targeted to improve resistance against eye infections [17]. The human ocular surface has been reported to have a self-sustaining microbiome composed largely of bacteria (98%), with fungi and viruses also present [18–20]. This resident microbial community imparts unique functions such as resistance to colonization by pathogens and modulation of intraocular immune and inflammation responses, thereby maintaining ocular health [17, 21, 22]. Perturbation of the ocular microbiome by factors such as dry eye disease, antimicrobials, infections, and contact lens usage can lead to dysbiosis, resulting in overgrowth of pathogens and intraocular inflammation [17, 23–25]. An altered ocular microbiome has been associated with many ophthalmic diseases in humans [26, 27]. Thus, restoring the homeostasis of the ocular surface microbiome to promote microbiome-mediated resistance against ophthalmic diseases is an active area of research in human medicine.

Although much progress has been made in investigating the compositional and functional features as well as the role of the ocular microbiome in human ocular health, the microbiome of the bovine eye remains less well characterized. This is partly due to the fact that the bovine ocular surface has traditionally been viewed as a habitat primarily for pathogenic microbes associated with IBK. However, a longitudinal study using 16S rRNA gene sequencing revealed the presence of a complex bacterial microbiota on the ocular surface of pre-weaned beef calves [28]. This study also demonstrated that the ocular microbiota in calves is dynamic, changing in composition, richness, and diversity in response to factors such as age, vaccination, and sampling practices. More recently, Gafen and colleagues identified reduced relative abundance of members of the Actinobacteriota phylum and greater relative abundance of *Moraxella* spp. in IBK-affected eyes compared to normal bovine eyes [29].

**Fig. 1:**
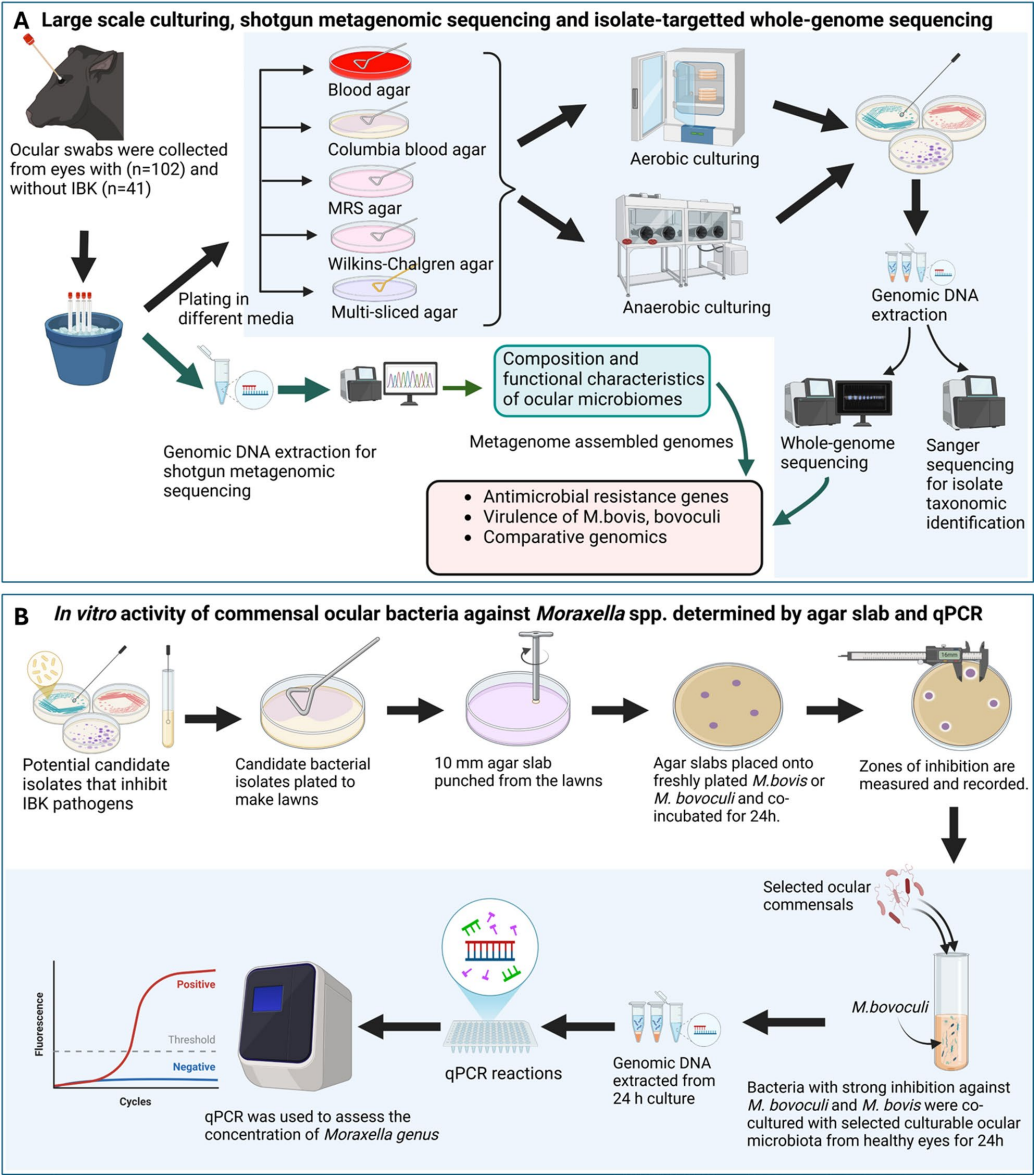
Schematic workflow diagram illustrating the experimental design, including the processes involved in ocular swab collection and processing, culture, shotgun metagenomic sequencing and isolate-targeted whole-genome sequencing (**A**), as well as the procedures used to screen isolated ocular commensal strains for their in vitro antimicrobial activity against *Moraxella bovis* and *Moraxella bovoculi* (**B**)

Our lab has also recently identified a relatively rich and site-specific bacterial community in the eyes of healthy newborn calves [30]. The genus *Moraxella* was well represented in those samples, and it is currently unclear whether early colonization of the eye with *Moraxella* spp. acts to prime the neonatal immune system against pathogens, or if it predisposes to infection. Fundamental questions also remain regarding how the ocular microbial composition, functional content, and antibiotic-resistance profiles differ between IBK-affected and healthy cattle, and whether commensal bacteria in the healthy ocular microbiome can inhibit *Moraxella* spp. pathogens. Therefore, the objectives of the present study were to: (1) characterize the ocular microbiome of healthy and IBK-affected beef cattle using culture-based methods and shotgun metagenomic sequencing techniques; (2) investigate the genetic diversity of *Moraxella bovis*, *Moraxella bovoculi*, and commensal ocular bacterial isolates through whole-genome sequencing and comparative genomic analysis; and (3) evaluate commensal ocular bacterial isolates for their ability to inhibit the growth of *Moraxella bovis* and *Moraxella bovoculi*, with the goal of enhancing resistance to IBK (Fig. 1).

## Materials and methods

All experimental procedures were approved by the North Dakota State University (NDSU; Fargo, ND, USA) Institutional Animal Care and Use Committee (protocol ID: 20220029).

### Ocular swab collection

Ocular swabs from the cornea and conjunctiva of cattle exhibiting IBK symptoms (*n* = 102) as well as control swabs from healthy animals (*n* = 41) were collected from multiple herds across North Dakota as well as from the NDSU Beef Cattle Research and Teaching Center and the NDSU Veterinary Diagnostic Laboratory (Table 1). Ocular swabs were collected from cattle diagnosed with IBK and healthy herd mates using a Puritan Opti-Swab with Liquid Amies Collection and Transport System (Puritan, Guilford, ME, USA). For swabbing, cattle were placed in a hydraulic squeeze chute and the head was manually restrained. Before swabbing, the eye was wiped with a clean paper towel sprayed with 70% ethanol to remove any debris. The eyelids were then opened, and both the conjunctiva and cornea were gently swabbed. Immediately after sample collection, the swab tips were broken off and placed in 1 mL of sterile Amies transport medium and stored on ice for transport to the lab. Upon arrival at the lab, the swab in Amies transport medium was vigorously vortexed, and a 150 µL aliquot of Amies medium was used for culturing. The remaining 850 µL of Amies medium and the swab were used for DNA extraction for shotgun metagenomic sequencing. For this, the tip was cut from the swab and placed into 1 mL of brain heart infusion (BHI) broth containing 20% glycerol. The Amies medium was then centrifuged (4,000 x *g* for 10 min), and 80% of the supernatant was discarded. The pellet was resuspended with the remaining supernatant, transferred to the BHI-glycerol tube containing the rayon tip of the swab, vortexed for 30 s, and stored at -80 °C until DNA extraction. As outlined in Fig. 1, the ocular swab samples collected from IBK-affected and healthy cattle were subjected to culturing and shotgun metagenomic sequencing.

**Table 1:** Summary of the ocular swab origins and bacterial isolates recovered from the ocular swab samples using culture-based methods

Source	Four ND Veterinary Clinics, NDSU Beef Herd, NDSU VDL					No. of summer swabs				110
						No. of winter swabs				33
**Swab type**	**Total number of swabs**	**Aerobic isolates**				**Anaerobic isolates**				**Total isolate number**
		**MP**	**CB**	**Blood**	**MRS**	**MP**	**Blood**	**WC**	**CB**	
**Healthy**	41	30	61	30	43	14	11	74	18	281
**IBK**	102	41	37	108	67	12	10	80	22	377
**Subtotal**	143	71	98	138	110	26	21	154	40	658

ND: North Dakota; NDSU: North Dakota State University; VDL: veterinary diagnostic laboratory; IBK: infectious bovine keratoconjunctivitis; CB: Columbia blood agar; MRS: De Man, Rogosa, and Sharpe agar; MP: multi-slice agar; WC: Wilkins-Chalgren

### Large-scale bacterial culturing of the ocular microbiome

A 150 µL aliquot from the swab samples was mixed with 250 µL of sterile Dulbecco’s phosphate-buffered saline and spread onto five different agar plate types (Blood, Columbia Blood [CB], De Man, Rogosa and Sharpe [MRS], Wilkins-Chalgren [WC], and multi-slice agars), to support the growth of a wide range of bacteria. The multi-slice agar is a custom-developed culture system in which a single Petri dish is partitioned into four equal sections, each containing a different growth medium. This design enables simultaneous inoculation of a single sample across multiple media types within the same plate, conserving sample volume and reducing handling time and labor while maximizing recovery of diverse bacterial taxa. The agar plates were incubated both aerobically and anaerobically for 24–48 h at 37 °C. The aerobic plates were supplemented with either 5% or 10% CO_2_ (MRS plate). Well-isolated colonies of different morphologies were selected and streaked onto fresh agar and incubated for 24 h. These bacterial isolates were then cryopreserved for DNA extraction and future cultivation by transferring a loopful of each isolate into 100 µL of TE buffer and 1 mL of BHI with 20% glycerol, respectively, and then stored at -80 °C.

A subset of the preserved bacterial isolates (*n* = 351) was identified by near full-length 16S rRNA gene sequencing. Isolates for taxonomic identification were randomly selected to include all agar types and culturing conditions. Genomic DNA from these selected isolates was extracted using the Zymo Quick-DNA Fungal/Bacterial Miniprep Kit (Zymo Research, Irvine, CA, USA) according to the manufacturer’s instructions with minor modifications as described previously [31]. The universal bacterial primers 27 F (5′ -AGAGTTTGATCMTGGCTCAG − 3′) [32] and 1492R (5′ - TACGGYTACCTTGTTACGACTT − 3′) [33] were used to amplify the near full-length 16S rRNA gene. Each PCR contained 20 µL of Phusion High-Fidelity DNA Polymerase (Thermo Fisher Scientific, Waltham, MA, USA), 12.8 µL of sterile water, 2 µL of each primer (10 µM) (IDT Inc., Coralville, IA, USA), 2 µL of isolate DNA, and 1.2 µL of DMSO (Thermo Fisher Scientific) in a total reaction volume of 40 µL. An Eppendorf Mastercycler thermocycler (Eppendorf, Hamburg, Germany) was then used with the following cycle conditions: initial denaturation at 95 °C for 5 min; 35 cycles at 95 °C for 45 s, 50 °C for 30 s, 72 °C for 2 min; and a final extension at 72 °C for 5 min. The PCR products were visualized on a 1% (w/v) agarose gel and the amplicons were sent to MCLAB (San Francisco, CA, USA) for Sanger sequencing. The resulting 16S rRNA gene sequences were identified using the Basic Local Alignment Search Tool (BLAST) and the non-redundant NCBI nucleotide database.

### Metagenomic DNA extraction from ocular swabs

A subset of 49 ocular swabs was subjected to shotgun metagenomic sequencing. For this, metagenomic DNA was extracted from the ocular swab samples using a modified cetyltrimethylammonium bromide buffer (CTAB) and phenol: chloroform-based method as described previously [34–36]. Briefly, samples were thawed on ice, vigorously vortexed, and the liquid portion was transferred into individual sterile 2 mL screw-cap tubes. Samples were then pelleted via centrifugation at 20,000 × *g* for 10 min at 22 °C. The supernatant was carefully removed, and the associated swab tip was snipped into the screw-cap tube containing the pellet. The beads from a Lysing Matrix E tube (MP Biomedicals, Irvine, CA, USA) were added to the sample tubes and 500 µL of CTAB buffer (10% CTAB, 0.7 M NaCl, 240 mM potassium phosphate buffer pH 8.0), prewarmed to 60 °C, was transferred to the sample tube and vortexed. Samples were then incubated for 20 min at 65 °C with agitation at 800 rpm, followed by further lysis using bead beating at 5.5 m/s for 30 s. Next, 500 µL of room temperature phenol: chloroform: isoamyl alcohol (25:24:1) was added and mixed by inversion.

The samples were centrifuged at 14,000 × *g* for 5 min, and the supernatant was transferred to a heavy phase-lock gel tube (Quantabio, Beverly, MA, USA). Chloroform was added to the tube at a 1:1 ratio, inverted to mix, and centrifuged at 14,000 × *g* for 5 min. The aqueous layer was transferred to a sterile 1.5 mL tube with 1 µL of linear acrylamide solution and vortexed. Two volumes of PEG-NaCl solution were then added, the tube was vortexed again, and then incubated for 2 h at 21 °C. After incubation, the samples were centrifuged at 14,000 × *g* for 30 min, the supernatant was carefully removed, and the resulting DNA pellet was washed with 400 µL of 70% ethanol and centrifuged at 14,000 × *g* for 5 min. This wash step was repeated twice. After the final centrifugation, the ethanol was removed and the pellet air dried for approximately 10 min. The DNA pellet was resuspended in 30 µL of 10 mM Tris-Cl (pH 8.0) and quantified using a NanoDrop One^C^ Microvolume UV-Vis Spectrophotometer (Thermo Fisher Scientific) followed by further quantification using the Quant-iT PicoGreen dsDNA Assay Kit (Thermo Fisher Scientific). DNA was then stored at -20 °C until shotgun metagenomic library preparation.

### Shotgun metagenomic sequencing

Metagenomic DNA libraries were constructed according to the following workflow. DNA was fragmented with a Covaris (E220) instrument (Covaris, Inc., Woburn, Massachusetts, USA) and the quality was assessed using an Agilent 2100 Bioanalyzer system (Agilent Technologies, Santa Clara, CA, USA). End-repair and dA-tailing were then carried out, followed by magnetic bead purification. Next, the fragments were PCR-amplified using KAPA HiFi HotStart DNA Polymerase, and the products were purified. The libraries were assessed for quality prior to DNA circularization. After circularization, the libraries were further amplified to create DNA nanoballs (DNB). Finally, the libraries were sequenced using DNA-based nanoball technology DNBSEQ on a DNBSEQ-G50 platform (MGI Tech, Shenzhen, China) with a PE150 flow cell and 2 × 150 bp sequencing length.

### Shotgun metagenomic data analysis

Sequencing adapters were removed from the raw sequences, and low-quality reads were filtered out using a 4-bp sliding window and a quality threshold of 15 in fastp v.0.23.4 [37]. Reads that were shorter than 100 bp were also removed. Bowtie2 v. 2.5.3 [38] was used to align the reads to the *Bos taurus* genome (ARS-UCD2.0) and the *Escherichia* phage phiX174 genome (NC_001422). SAMtools v.1.19.2 [39] and BEDtools v.2.31.1 [40] were then used to obtain the reads that did not map to these genomes. Kraken2 v. 2.1.3 [41] and Bracken v. 2.9 [42], together with the Genome Taxonomy Database (GTDB) release 220 [43], were used to assign taxonomy to the metagenomic reads. The unassembled metagenomic reads were also screened for antimicrobial resistance genes (ARGs) using the Resistance Gene Identifier (RGI) v. 6.0.3 and the Comprehensive Antibiotic Resistance Database (CARD) v.3.2.9 (23) with KMA [44].

Reads from each sample were assembled individually and reads from IBK-affected and healthy cattle samples were co-assembled separately using MEGAHIT v. 1.2.9 [45]. The reads from each sample were then aligned to the assembled contigs using Bowtie2 and the contigs were then binned into metagenome-assembled genomes (MAGs) with MetaBAT2 v. 2.15 [46]. The completeness and contamination of these MAGs were evaluated with CheckM2 v. 1.0.1 [47] and MAGs with a completeness of at least 90% and a contamination level of less than 5% were retained. These MAGs were then dereplicated using dRep v. 3.4.5 [48] with primary clustering set at 90% average nucleotide identity (ANI) and secondary clustering set at 99% ANI. Taxonomy was then assigned to the dereplicated MAGs using GTDB-tk v. 2.4.0 [49]. The MAGs were also screened for ARGs using the RGI and CARD. The relative abundance of the MAGs in the metagenomes was estimated using CoverM v. 0.7.0 (github.com/wwood/CoverM). Functional gene content was characterized by aligning the metagenomic reads to the Kyoto Encyclopedia of Genes and Genomes (KEGG) [50] prokaryotic protein database (release 112.0) with DIAMOND v. 2.1.8.162 in blastx mode [51] Reads were assigned to KEGG orthology (KO) groups and then aggregated to KEGG pathways.

### DNA extraction from ocular commensal and *Moraxella* spp. isolates

Selected *Moraxella bovis* (*n* = 5) and *Moraxella bovoculi* (*n* = 13) isolates as well as potential candidate bacterial isolates (*n* = 13) for inhibition of the growth of *Moraxella* spp. were subjected to whole-genome sequencing. For this, the isolates were re-grown from BHI-glycerol stocks on blood or MRS agar and passaged twice before a single colony was transferred to 5 mL of BHI or MRS broth and incubated for 24 h at 37 °C with 5 or 10% CO_2_. Genomic DNA was extracted from the isolates using the DNeasy Blood and Tissue Kit (Qiagen Inc.). Briefly, 1 mL of 24-h isolate culture was centrifuged at max speed (20,817 × *g*) for 5 min at 21 °C to pellet the bacterial cells. To increase the mass of the pellet recovered, this step was repeated up to three times. To the cell pellet, 180 µL of enzymatic lysis buffer (20 mM Tris-HCl, pH 8, 2 mM sodium EDTA, 1.2% Triton X-100, lysozyme [100 mg/mL], and mutanolysin [25,000 U/mL]) was added and thoroughly vortexed. Samples were incubated for 1 h at 37 °C with agitation at 800 rpm. This incubation was followed by the addition of 25 µL of proteinase K and 200 µL of buffer AL (without added ethanol), thorough vortexing of samples, and a 30-min incubation at 56 °C with agitation at 800 rpm. After the second incubation, samples were mechanically lysed by adding approximately 400 mg of sterile 0.1-mm zircon/silica beads to each tube and bead beating at 6.0 m/s for 40 s using a FastPrep-24 Classic bead beater (MP Biomedicals). Samples were then centrifuged at 13,000 × *g* for 5 min, and the supernatant was transferred to a new sterile 1.5 mL tube. The remaining steps were followed according to the manufacturer’s instructions.

### Whole-genome sequencing and analysis

Whole-genome sequence libraries for the ocular commensal and *Moraxella* spp. isolates were prepared and sequenced as we described in our previous publication [52]. Whole-genome sequences from bacterial isolates were quality-filtered using SOAPnuke v. 1.5.6, with adapters and sequences shorter than 150 bp or with a quality threshold of less than 20 removed. The reads were assembled using SPAdes v. 3.15.5 with the “isolate” option and the assembly quality was evaluated using QUAST v. 5.2.0. The assemblies were then taxonomically classified using GTDB-Tk v. 2.4.0 and GTDB release 220. The vast majority of the genomes had 500X or greater coverage, which may affect the quality of the assembly. Therefore, the reads were subsampled to 100X for each genome using seqtk v. 1.4-r122 (github.com/lh3/seqtk) and re-assembled with SPAdes. The completeness and contamination of each 100X assembly were then assessed with CheckM2 v. 1.0.1. A phylogenetic tree of the bacterial genomes as well as MAGs from the metagenomes was created using PhyloPhlAn v. 3.1.68 [53] and visualized in iTol v. 6.9 [54]. FastANI was used to determine which isolates might belong to the same strain (99.99% shared ANI). The genomes were also screened for ARGs using the RGI and CARD and mapped against the metagenomic reads with CoverM to assess their abundance in the ocular metagenomes.

### Comparative genomic analysis of *Moraxella bovis* and *Moraxella bovoculi*

Given their association with IBK, the *Moraxella bovis* and *Moraxella bovoculi* MAGs and isolate genomes were further analyzed. Briefly, the *Moraxella* spp. genomes and MAGs were annotated using Prokka v. 1.14.6 [55] with default parameters and the proteins flag using the reference genomes in GenBank for *Moraxella bovis* (GCA_025985205.1) and *Moraxella bovoculi* (GCA_000988745.3). After annotation, Panaroo v. 1.5.0 [56] was used to determine the core genome for each species using the strict stringency mode. The core genes (0.99 threshold) were aligned with MAFFT v. 7.526 [57] and a phylogenetic tree was created from the core genome alignment with RAxML-ng v.1.2.0 [58] for each species with 1,000 bootstrap 1,000 replicates and the GTR+GAMMA substitution model.

The *Moraxella* spp. MAGs and genomes were also screened for known virulence genes with DIAMOND with a 90% identity threshold. These virulence genes included the RTX operon genes in *Moraxella bovis* designated *mbxA* (AAK84651.1), *mbxB* (AAP74653.1), *mbxC* (AAP74652.1), and *mbxD* (AAP74654.1) as well as the *Moraxella bovoculi* RTX operon genes *mbvA* (AKM27892.1), *mbvB* (ABA42642.1), *mbvC* (ABA42640.1), and *mbvD* (ABA42643.1). Additional virulence genes assessed were *flpA* (filamentous-haemagglutinin-like protein; BAD83731.1), *flpB* (filamentous-haemagglutinin-like protein; BAD83735.1), *fur* (ferric uptake regulator; BAC75975.1; KDN24651.1), *omp79* (outer membrane protein; BAC92729.1), *pilA* (type IV pilin; QPW18723.1; STY93141.1), *plb* (phospholipase B; AAK53448.1), and *tolC* (outer membrane protein; AAP74655.2; ABA42644.1). Plasmids and MOB genes were identified in the genome assemblies using MOB-suite v. 3.1.9 [59].

### In vitro antimicrobial activity of ocular commensal bacteria against *Moraxella* spp.

Of the 351 isolates selected for identification, 52 were tested for growth-inhibitory effects against *Moraxella bovis* and *Moraxella bovoculi* using the agar slab method, as previously described [60] (Fig. 1B). Briefly, to visualize bacterial antimicrobial activity, a small agar slab (10 mm in diameter) containing the bacterial isolate of interest was placed on the surface of an agar plate with a fresh inoculum of *Moraxella bovis* (8338-1) or *Moraxella bovoculi* (42G CB-D strain). For this, 100 µL of a 24-h culture was spread as a lawn onto BHI or MRS agar plates and incubated for 24 h at 37 °C with 5 or 10% CO_2_. From these 24-h bacterial lawns, a 10-mm sterile cork borer was used to cut agar slabs. The agar slabs were placed surface-side down onto a fresh lawn of *Moraxella bovis* or *Moraxella bovoculi* on TSA plates supplemented with 5% sheep’s blood. The *Moraxella bovis* and *Moraxella bovoculi* plates were prepared by spreading 100 µL of *Moraxella bovis* or *Moraxella bovoculi* overnight culture onto the plates and drying for up to 30 min at 37 °C with 5% CO_2_. Up to four agar slabs were placed on the *Moraxella* lawns and a control agar slab that contained no bacteria was included in each screening. After co-incubation for 24 h at 37 °C with 5% CO_2_, inhibition of *Moraxella bovis* or *Moraxella bovoculi* growth was observed using the zone of inhibition (ZOI) surrounding the agar plug containing the bacteria of interest. The diameter of the ZOI was measured using a digital caliper and reported as the mean diameter. *Limosilactobacillus fermentum* ATCC9338 was also included in the screening for comparison.

### Growth-inhibitory effects of selected ocular commensal bacteria against *Moraxella bovoculi* in the presence of the ocular microbiome

Following the agar slab experiment, a selection of candidate bacteria that exhibited relatively strong inhibition of *Moraxella* growth were further evaluated for their ability to inhibit the growth of *Moraxella* spp. in the presence of the culturable ocular microbiome. *Moraxella bovoculi* was first co-incubated with selected commensal isolates in an enriched ocular microbiome culture. The enriched culture of the ocular microbiome was prepared by pooling 100 µL from five healthy ocular swabs cryopreserved in BHI and 20% glycerol and adding 2.5 mL of fresh BHI broth. This mixture was then incubated at 37 °C for approximately 20 h with agitation at 200 rpm.

Next, 60 µL of this enriched ocular microbiome was combined with 60 µL of *Moraxella bovoculi* overnight culture, 60 µL of overnight culture of the selected commensal bacterial isolate, and 2.82 mL of fresh BHI broth, for a total volume of 3 mL. Each candidate bacterial strain co-culture was run in quadruplicate. In addition, a cocktail containing all candidate strains was included. Controls included: (1) only the enriched ocular microbiome and BHI broth, (2) only *Moraxella bovoculi* and BHI broth, and (3) only BHI broth. Prior to incubation, 200 µL of the prepared cultures were saved as baseline (0 h) samples. Cultures were incubated for 24 h at 37 °C with agitation at 200 rpm. After incubation, 200 µL from each replicate was used for 24 h samples. DNA from both 0 h and 24 h samples was extracted using the same procedure described above for whole-genome sequencing.

Quantitative PCR (qPCR) was used to assess the concentration of *Moraxella* spp. in each sample to observe whether the selected commensal strains could inhibit *Moraxella* growth in the presence of the bovine ocular microbiome. The primers ISRdown (5′-GTGAAGTCGTAACAAGGTAGCCGT-3′) and ISRup (5′-ACCGACGCTTATCGCAGGCTATCA-3′) [61] were used to amplify the 16–23 S rRNA intergenic spacer region (ISR). Each qPCR mixture contained 10 µL SsoAdvanced Universal SYBR Green Supermix (Bio-Rad Laboratories, Inc., Hercules, CA, USA), 1 µL of each primer (IDT Inc.), 6.8 µL of molecular biology grade water (Corning, Manassa, VA, USA), and 1.2 µL of DNA template, in a total volume of 20 µL. A CFX96 Touch Real-Time PCR Detection system (Bio-Rad Laboratories Ltd.) was used with the following conditions: an initial denaturation at 98 °C for 3 min, followed by 39 cycles at 98 °C for 15 s, 60 °C for 1 min, and then a melt curve analysis was performed starting at 65 °C for 5 s, with a 0.5 °C increase per cycle, ending at 95 °C. Standard curves (10^2^ to 10^9^ gene copies) were produced using the pDrive cloning vector (Qiagen Inc.) containing the PCR product from the 16–23 S rRNA ISR [11]. Each standard was run in duplicate and a no-template control (1.2 µL of nuclease-free water) was included.

### Statistical analysis

Permutational multivariate analysis of variance (PERMANOVA) was performed on Bray-Curtis dissimilarity calculated from the relative abundance data (total sum scaling) for archaeal and bacterial species and from copies per million reads for KEGG pathways using the vegan package (v. 2.6-4) in R to assess the effect of IBK status on the ocular surface microbiome. The Mann-Whitney test was used to compare the inverse Simpson diversity index values and qPCR results between IBK-affected and healthy cattle as the data were not normally distributed. Differentially abundant ARGs, microbial species, and KEGG pathways in the ocular microbiomes of IBK-affected vs. healthy cattle were identified using MaAsLin2 v. 1.16.0 (Mallick, et al. 2021) in R v. 4.3.2. Only ARGs and KEGG pathways found in at least 25% of the samples, and microbial species with an overall relative abundance of ≥ 0.1%, were included for the differential abundance analyses.

## Results

### Summary of the ocular swabs collected

A total of 143 ocular swabs were collected: 102 from cattle diagnosed with IBK and 41 from healthy cattle. The majority of the ocular swabs (77%) were collected during the summer, while additional samples were obtained from winter IBK outbreaks (Table 1).

### The ocular microbiome assessed by large-scale culturing

Through extensive aerobic and anaerobic culturing using 5 different agar types, 658 isolates were recovered (data not shown). The majority of these isolates (*n* = 417) were recovered under aerobic conditions. In addition, 58% of the isolates were obtained from culture of swabs from IBK-affected cattle, although this was expected given that a larger number of swabs were collected from IBK-affected cattle. Overall, the highest numbers of isolates were recovered from WC (anaerobic, *n* = 154), blood (aerobic, *n* = 138), and MRS (aerobic, *n* = 110) agar plates. Of the 658 bacterial isolates collected, 351 were selected for taxonomic identification via Sanger sequencing of the near full-length 16S rRNA gene. These isolates represented three bacterial phyla: Bacillota (81.8%), Pseudomonadota (13.4%), and Actinomycetota (4.8%) (Table 2). Among the 61 bacterial genera identified, isolates belonging to *Bacillus* (26%), *Streptococcus* (11%), *Staphylococcus* (11%), *Moraxella* (9%), and *Macrococcus* (4%) were most frequently recovered (Table 2). Thirty-three *Moraxella* isolates obtained, consisting of both *Moraxella bovis* and *Moraxella bovoculi.*

**Table 2:** Bacterial isolates (*n* = 351) recovered and identified from aerobic and anaerobic culturing of ocular swab samples obtained from IBK-affected and healthy cattle

Phylum	Genus	Number of isolates	Percentage of total isolates	Total phylum prevalence
Actinomycetota	*Corynebacterium*	4	1.1%	4.8%
Actinomycetota	*Trueperella*	2	0.6%	
Actinomycetota	*Luteococcus*	2	0.6%	
Actinomycetota	*Arthrobacter*	2	0.6%	
Actinomycetota	*Cellulosimicrobium*	1	0.3%	
Actinomycetota	*Streptomyces*	1	0.3%	
Actinomycetota	*Rothia*	1	0.3%	
Actinomycetota	*Nocardia*	1	0.3%	
Actinomycetota	*Cutibacterium*	1	0.3%	
Actinomycetota	*Curtobacterium*	1	0.3%	
Actinomycetota	*Micrococcus*	1	0.3%	
Bacillota	*Bacillus*	91	25.9%	81.8%
Bacillota	*Streptococcus*	39	11.1%	
Bacillota	*Staphylococcus*	39	11.1%	
Bacillota	*Macrococcus*	14	4.0%	
Bacillota	*Paenibacillus*	9	2.6%	
Bacillota	*Caldibacillus*	9	2.6%	
Bacillota	*Rummeliibacillus*	8	2.3%	
Bacillota	*Aerococcus*	8	2.3%	
Bacillota	*Desemzia*	7	2.0%	
Bacillota	*Mammaliicoccus*	5	1.4%	
Bacillota	*Weizmannia*	4	1.1%	
Bacillota	*Niallia*	4	1.1%	
Bacillota	*Heyndrickxia*	4	1.1%	
Bacillota	*Carnobacterium*	4	1.1%	
Bacillota	*Alkalihalobacillus*	4	1.1%	
Bacillota	*Robertmurraya*	3	0.9%	
Bacillota	*Priestia*	3	0.9%	
Bacillota	*Enterococcus*	3	0.9%	
Bacillota	*Cytobacillus*	2	0.6%	
Bacillota	*Brevibacillus*	2	0.6%	
Bacillota	*Peribacillus*	2	0.6%	
Bacillota	*Lysinibacillus*	2	0.6%	
Bacillota	*Lentilactobacillus*	2	0.6%	
Bacillota	*Helcococcus*	2	0.6%	
Bacillota	*Weissella*	1	0.3%	
Bacillota	*Virgibacillus*	1	0.3%	
Bacillota	*Terribacillus*	1	0.3%	
Bacillota	*Ruoffia*	1	0.3%	
Bacillota	*Planococcus*	1	0.3%	
Bacillota	*Peptoniphilus*	1	0.3%	
Bacillota	*Paraclostridium*	1	0.3%	
Bacillota	*Oceanobacillus*	1	0.3%	
Bacillota	*Levilactobacillus*	1	0.3%	
Bacillota	*Lactiplantibacillus*	1	0.3%	
Bacillota	*Lacrimispora*	1	0.3%	
Bacillota	*Globicatella*	1	0.3%	
Bacillota	*Fundicoccus*	1	0.3%	
Bacillota	*Faecalicatena*	1	0.3%	
Bacillota	*Facklamia*	1	0.3%	
Bacillota	*Exiguobacterium*	1	0.3%	
Bacillota	*Clostridium*	1	0.3%	
Pseudomonadota	*Moraxella*	33	9.4%	13.44%
Pseudomonadota	*Suttonella*	4	1.1%	
Pseudomonadota	*Mannheimia*	3	0.9%	
Pseudomonadota	*Acinetobacter*	2	0.6%	
Pseudomonadota	*Psychrobacter*	1	0.3%	
Pseudomonadota	*Pseudoxanthomonas*	1	0.3%	
Pseudomonadota	*Pantoea*	1	0.3%	
Pseudomonadota	*Citrobacter*	1	0.3%	
Pseudomonadota	*Alysiella*	1	0.3%	
**Total**		**351**	**100%**	

### Metagenomic sequencing summary

The ocular surface microbiome was also characterized using shotgun metagenomic sequencing. The vast majority of the metagenomic reads were derived from the host genome: 96.4 ± 1.0% (SEM) for samples from healthy cattle and 92.9 ± 1.9% for samples from IBK-affected cattle. After host DNA removal, the average read counts for the healthy and IBK-affected cattle samples were 2,575,944 ± 791,376 and 4,312,305 ± 1,074,688, respectively.

### Ocular microbiome of IBK-affected vs. healthy cattle

**Fig. 2:**
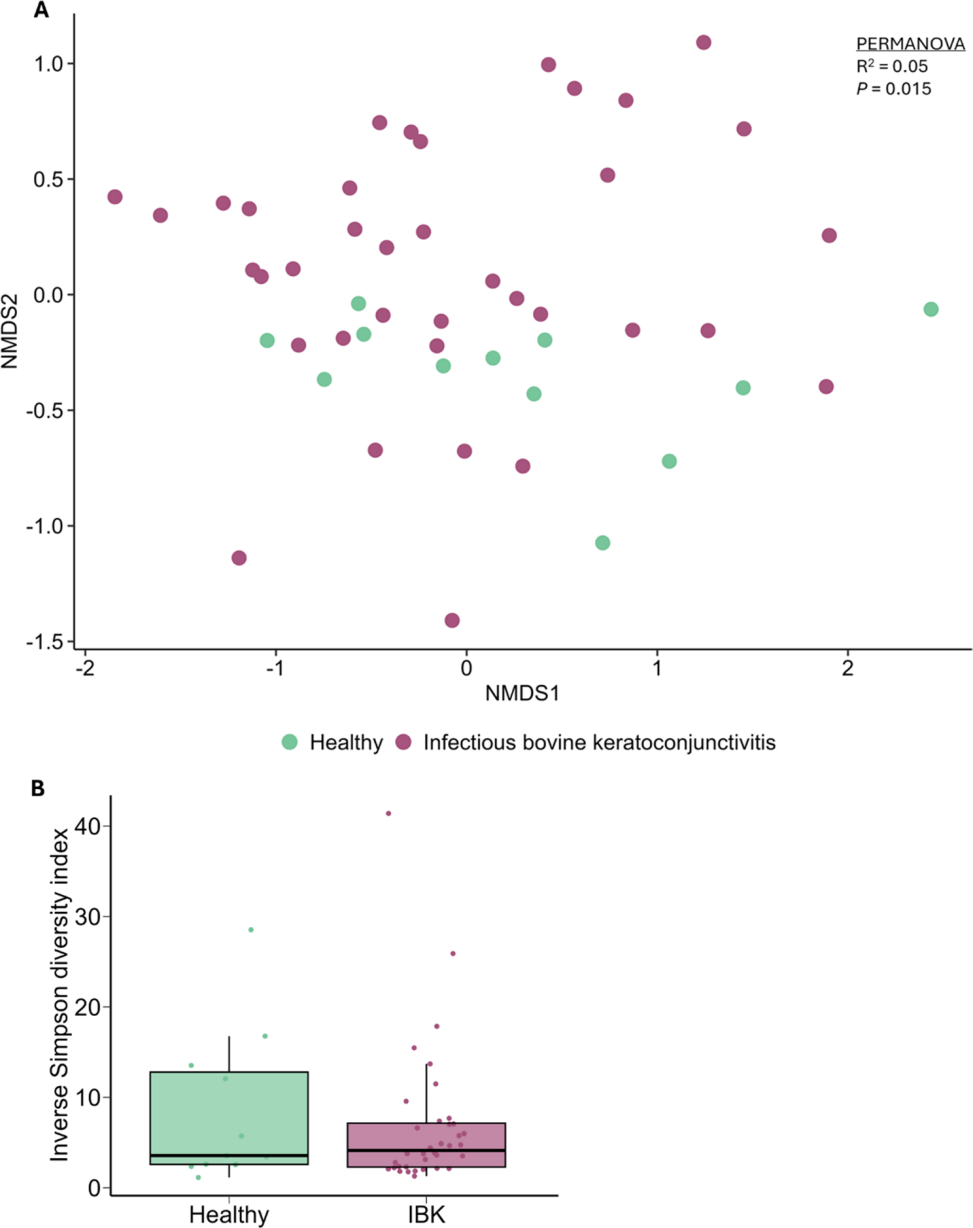
A non-metric multidimensional scaling (NMDS) plot based on Bray Curtis dissimilarities (**A**), and inverse Simpson diversity index (**B**) for the ocular microbiome of healthy cattle (*n* = 12) and cattle diagnosed with infectious bovine keratoconjunctivitis (IBK) (*n* = 37; *P* = 0.54)

**Fig. 3:**
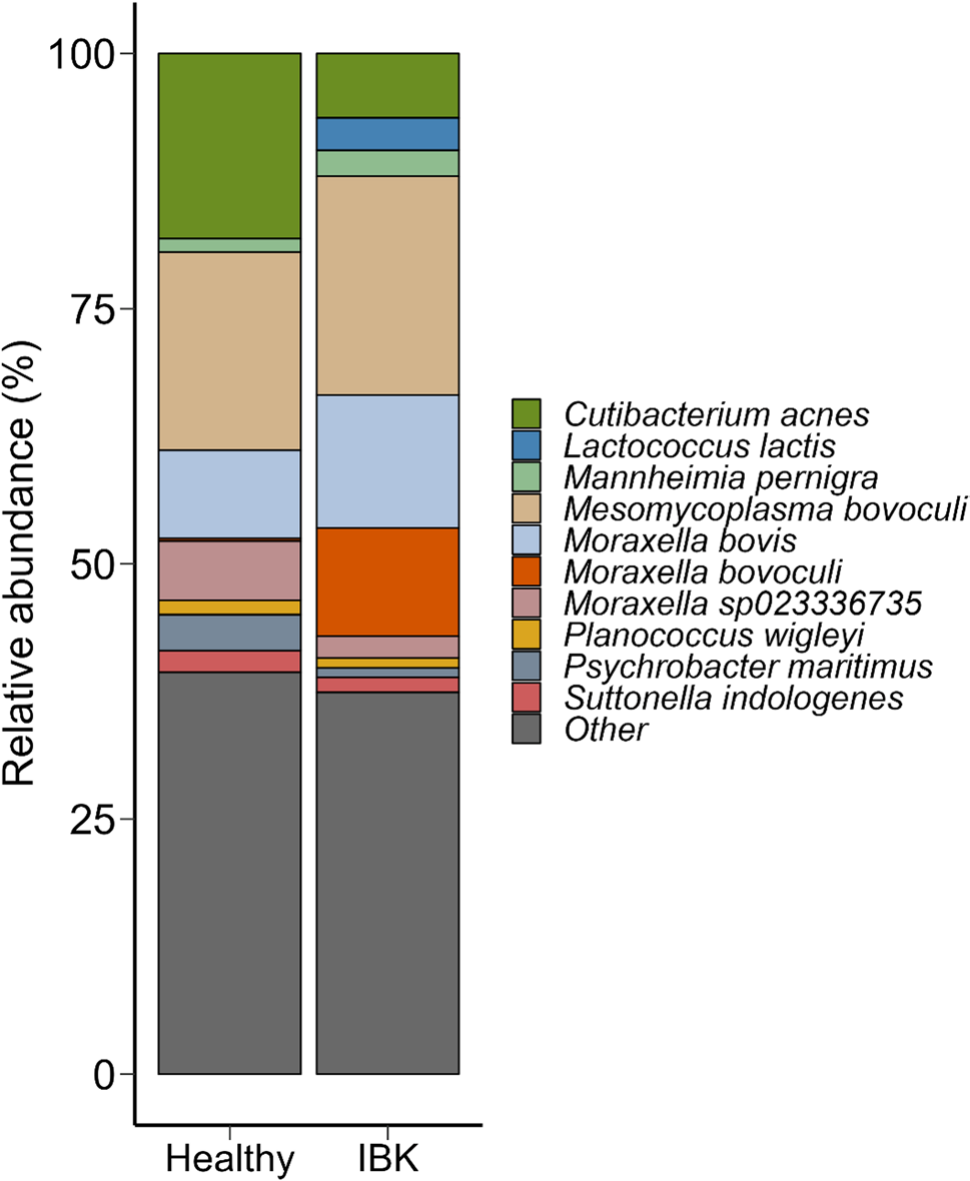
Stacked bar chart of the 10 relatively most abundant bacterial species in the ocular surface microbiomes of healthy cattle (*n* = 12) and cattle diagnosed with infectious bovine keratoconjunctivitis (IBK; *n* = 37)

**Fig. 4:**
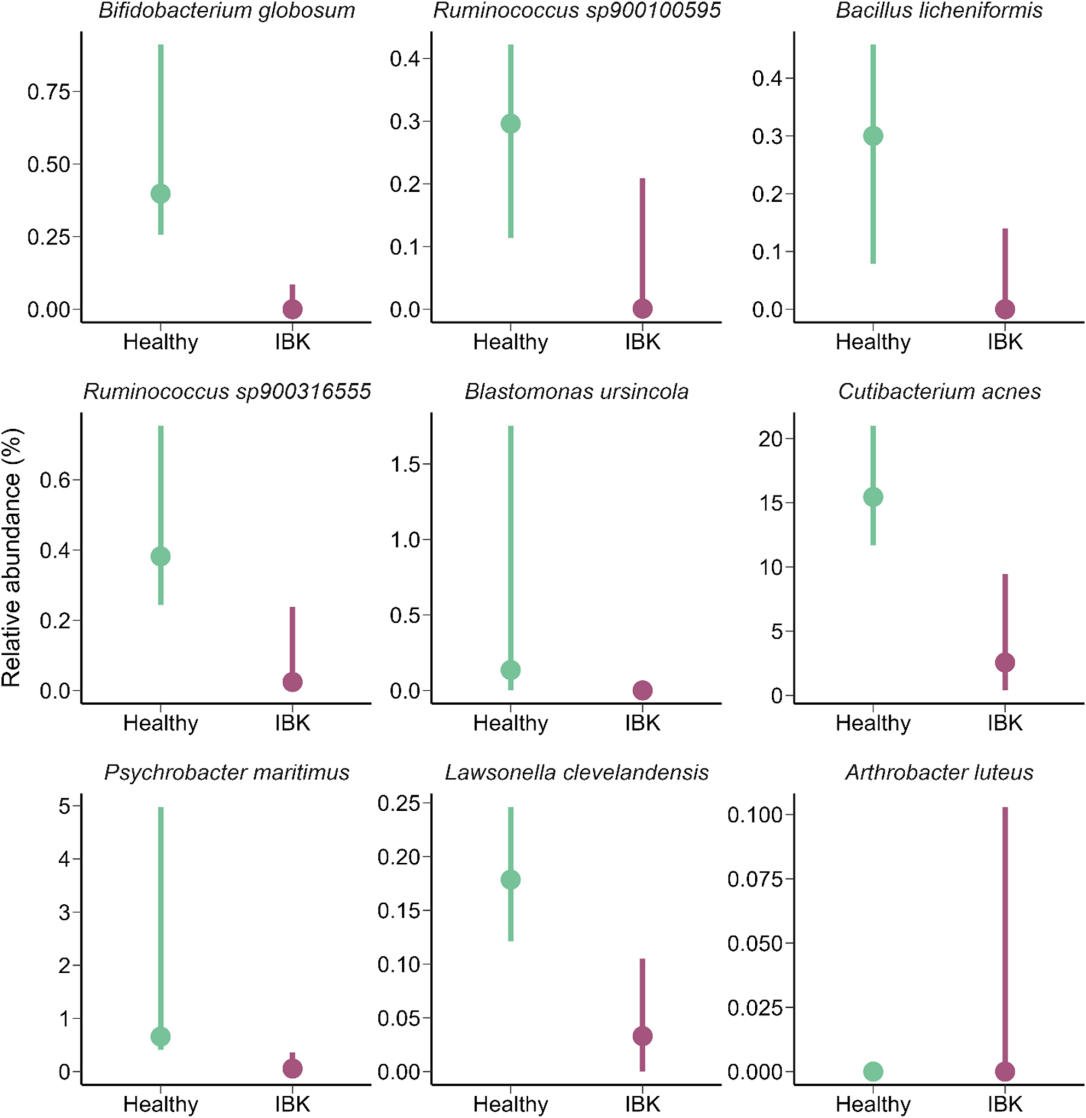
Percent relative abundance of differentially abundant bacterial species in the ocular surface microbiomes of healthy cattle (*n* = 12) and cattlediagnosed with infectious bovine keratoconjunctivitis (IBK) (*n* = 37; *P* < 0.05). Points represent the median value and lines are the 25th and 75th percentiles

There was a statistically significant (PERMANOVA: *P* = 0.015) but modest difference (*R*^*2*^ = 0.05) in the ocular surface microbial species composition between healthy cattle and cattle diagnosed with IBK based on Bray-Curtis dissimilarity (Fig. 2A). Microbial species diversity on the ocular surface did not differ between healthy and IBK-affected cattle (Fig. 2B). Overall, the ocular surface microbiomes were dominated by bacterial species such as *Cutibacterium acnes*, *Mannheimia pernigra*, *Mesomycoplasma bovoculi*, *Moraxella bovis*, and *Moraxella bovoculi* (Fig. 3). Nine bacterial species were differentially abundant in the ocular microbiomes of healthy versus IBK-affected cattle (Fig. 4). All but one of these species (*Arthrobacter luteus*) were relatively more abundant in healthy cattle and included *Bacillus licheniformis*, *Bifidobacterium globosum*, *Blastomonas ursincola*, *C. acnes*, *Lawsonella clevelandensis*, *Psychrobacter maritimus*, *Ruminococcus* sp900100595, and *Ruminococcus* sp900316555.

**Fig. 5:**
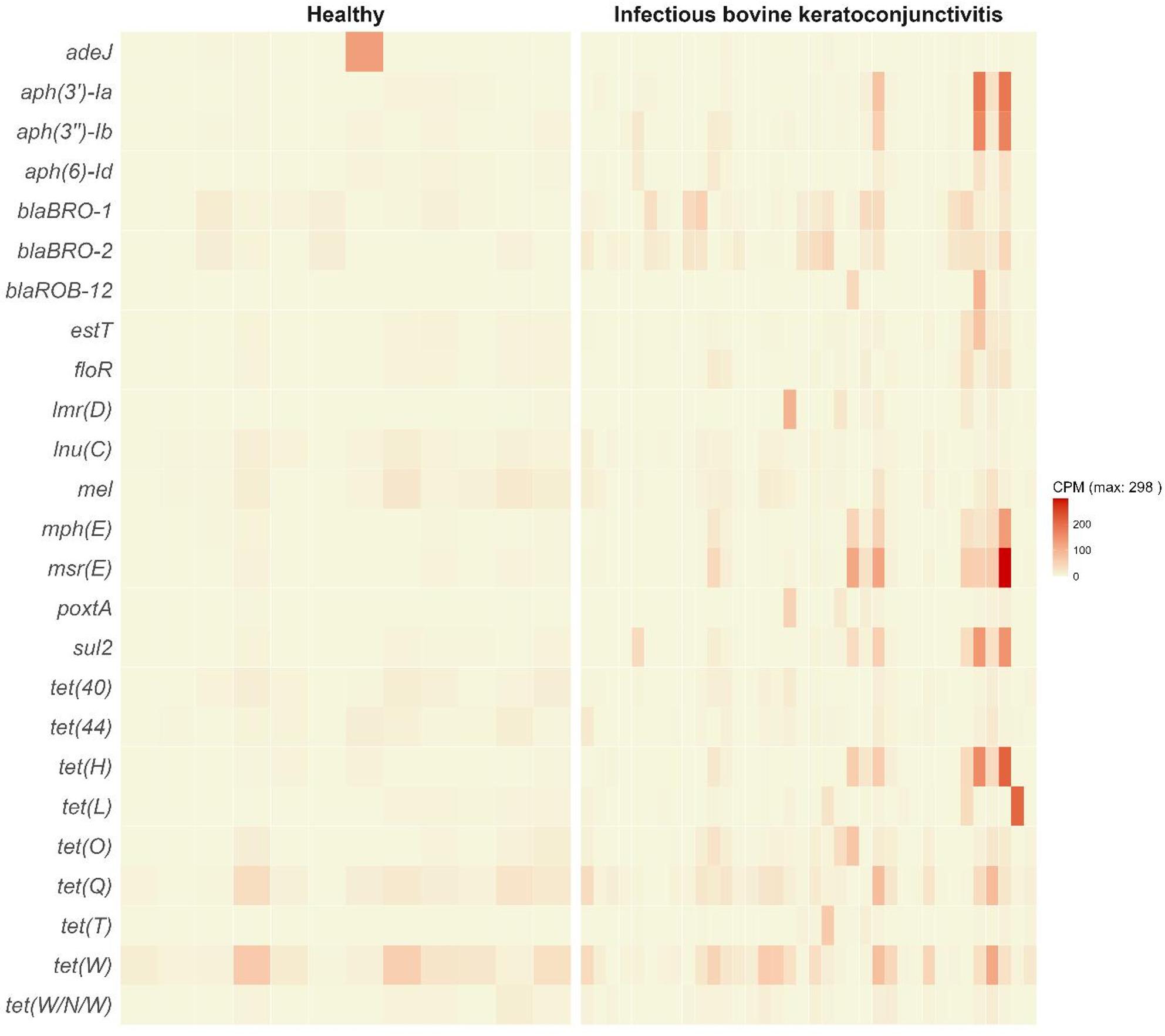
Heatmap of the 25 most abundant antimicrobial resistance genes identified in the ocular surface microbiomes of healthy cattle (*n* = 12) and cattle diagnosed with infectious bovine keratoconjunctivitis (*n* = 37). CPM: copies per million reads

The functional content of the ocular surface microbiome was assessed using KEGG pathways. In total, 158 KEGG pathways were identified in at least one ocular sample with ko01100 (metabolic pathways), ko01240 (biosynthesis of cofactors), and ko01230 (biosynthesis of amino acids) being the most abundant (data not shown). The overall functional composition did not differ between IBK-affected and healthy cattle (Fig. S1; PERMANOVA: *P* > 0.05). Similarly, no KEGG pathways were differentially abundant in the ocular microbiomes of IBK-affected versus healthy cattle (*P* > 0.05). Furthermore, none of the ARGs identified in the ocular metagenomes were differentially abundant between the two groups of cattle. Overall, ARGs conferring resistance to aminoglycosides [*aph(3’)-Ia*, *aph (3’’)-Ib*], macrolides [*mel*, *mph*(E), *msr*(E)], sulfonamides (*sul2*), tetracyclines [*tet*(H), *tet*(L), *tet*(O), *tet*(Q), and *tet*(W)] were most abundant, although considerable inter-individual variation was observed (Fig. 5).

### Metagenome-assembled genomes (MAGs)

**Fig. 6:**
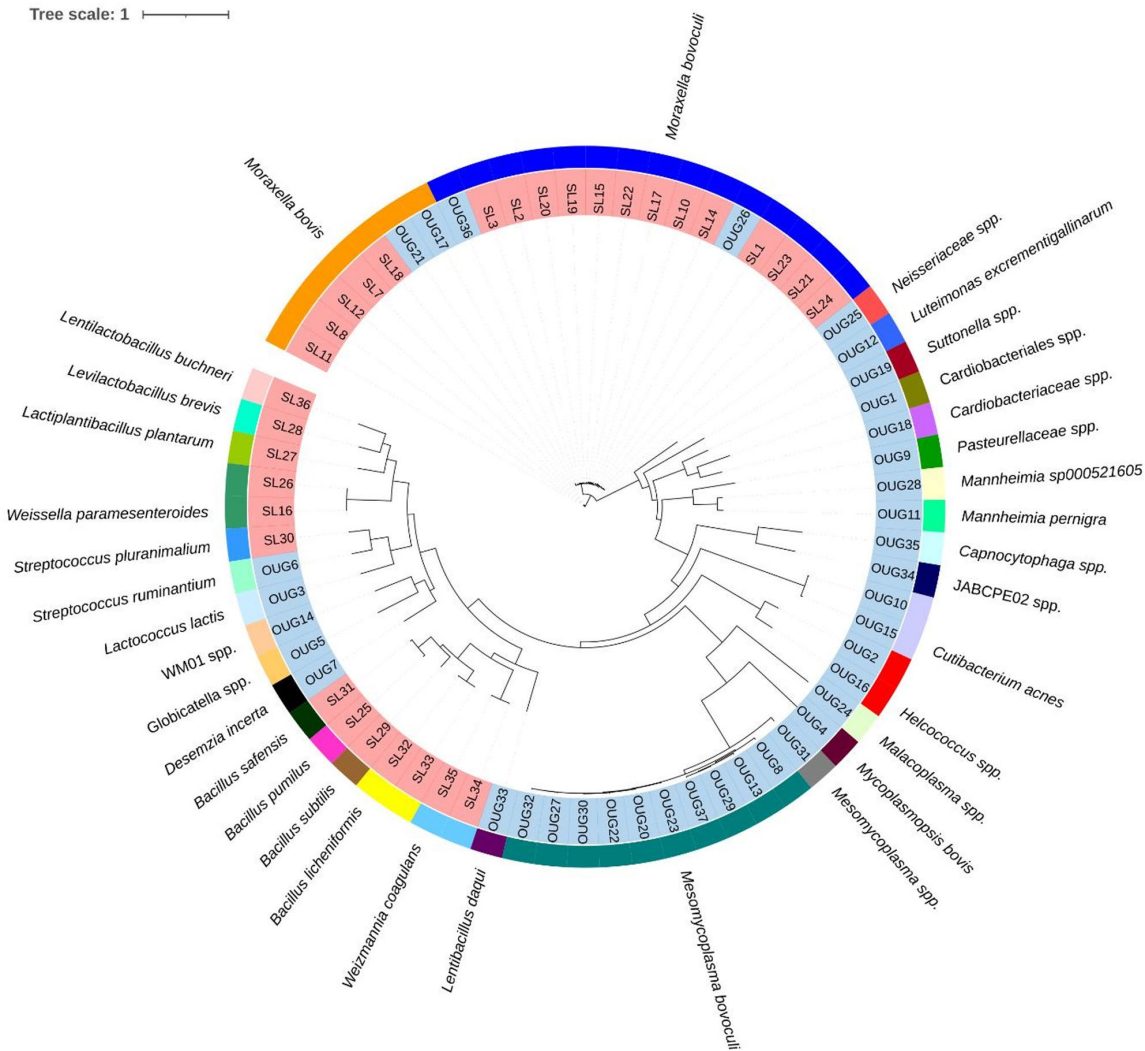
Maximum likelihood phylogenetic tree of 31 bacterial isolate genomes and 37 metagenome-assembled genomes (MAGs) from the ocular surface of cattle. Isolate genomes are shown in red and MAGs are shown in blue in the inner ring

There were also 37 dereplicated high-quality MAGs recovered from the ocular metagenomes, 12 of which appeared to represent novel species (Fig. 6, Supplementary Table S1). The most frequently recovered MAGs (*n* = 10) were identified as *Mesomycoplasma bovoculi*. Other identified species included *Moraxella bovis*, *Moraxella bovoculi*, *Luteimonas excrementigallinarum*, *M. pernigra*, *C. acnes*, *Mycoplasma bovis*, *Lentibacillus daqui*, *Desemzia incerta*, *Lactococcus lactis*, and *Streptococcus ruminantium*. The MAGs were also screened for ARGs and 13 MAGs carried at least one ARG, including *bla*_BRO−1_ in both *Moraxella bovis* MAGs and in *Moraxella bovoculi* OUG26 (Supplementary Table S1). Other ARGs of note that were identified included *floR* (phenicols) in *Moraxella bovoculi* OUG36 and *ant(9)-Ia* (aminoglycosides), *bla*III (beta-lactams), *erm*(A) (macrolides), f*exA* (phenicols), *msr*(I) (macrolides), and *vanG* (vancomycin) in *L. daqui* OUG33. Overall, the most relatively abundant MAG in the ocular surface metagenomes was MAG OUG1, which was taxonomically identified only at the order level (Cardiobacteriales). This MAG was also the only one with a significantly different relative abundance between the two groups of cattle (*P* < 0.05), although there was considerable inter-individual variability.

### Isolate-targeted whole-genome sequencing

The genomes of 31 bacterial isolates were sequenced, including 5 *Moraxella bovis* and 13 *Moraxella bovoculi* genomes (Supplementary Table S1). The remaining 13 genomes were ocular bacterial isolates that inhibited *Moraxella*, and included *Bacillus licheniformis* (*n* = 2), *W. coagulans* (*n* = 2), *Weissella paramesenteroides* (*n* = 2), *B. pumilus*, *Bacillus safensis*, *Bacillus subtilis*, *Levilactobacillus brevis*, *Lactiplantibacillus plantarum*, *Streptococcus pluranimalium*, and *L. buchneri.* All 5 *Moraxella bovis* isolates appeared to represent unique strains based on ANI values < 99.99% [62] while there were 10 *Moraxella bovoculi* strains based on this criterion. The *Bacillus licheniformis*, *W. coagulans*, and *W. paramesenteroides* isolates also appeared to be unique strains.

These 31 isolate genomes were also screened for the presence of ARGs. Four of the *Moraxella bovis* and two *Moraxella bovoculi* isolates carried the beta-lactamase gene *bla*_BRO−1_, which was originally described in *Moraxella catarrhalis *[63]. The tetracycline efflux pump gene *tet*(45) was found in two *Moraxella bovoculi* isolates (SL21, SL24) belonging to the same strain, as well as the *B*. *subtilis* SL29 isolate. Several other ARGs associated with *Bacillus* spp. were identified including the chloramphenicol acetyltransferase gene *cat86* in *B*. *pumilus* and *B*. *safensis*, the class D beta-lactamase *bla*_BPU−1_ in *B*. *pumilus*, and *fosBx1*, a thiol transferase gene conferring resistance to fosfomycin, in *B*. *licheniformis* and *B*. *subtilis*. When the metagenomic reads were mapped to the isolate genomes, the *Moraxella bovis* and *Moraxella bovoculi* genomes were most abundant, while many of the non-*Moraxella* spp. genomes were not detected, likely due to limited sequencing depth (Supplementary Table S2). Similarly, none of the non-*Moraxella* spp. isolates were assembled and binned into high-quality MAGs from the metagenomes. However, the *Moraxella bovis* OUG21 MAG shared an ANI of 99.94% with *Moraxella bovis* isolates SL11 and SL18 and *Moraxella bovoculi* MAG OUG26 and isolate SL1 had an ANI of 99.97%. Plasmids were reconstructed from several isolates including *Moraxella bovis* and *Moraxella bovoculi*; however, none of the ARGs detected were found to be carried on one of these plasmids.

**Fig. 7:**
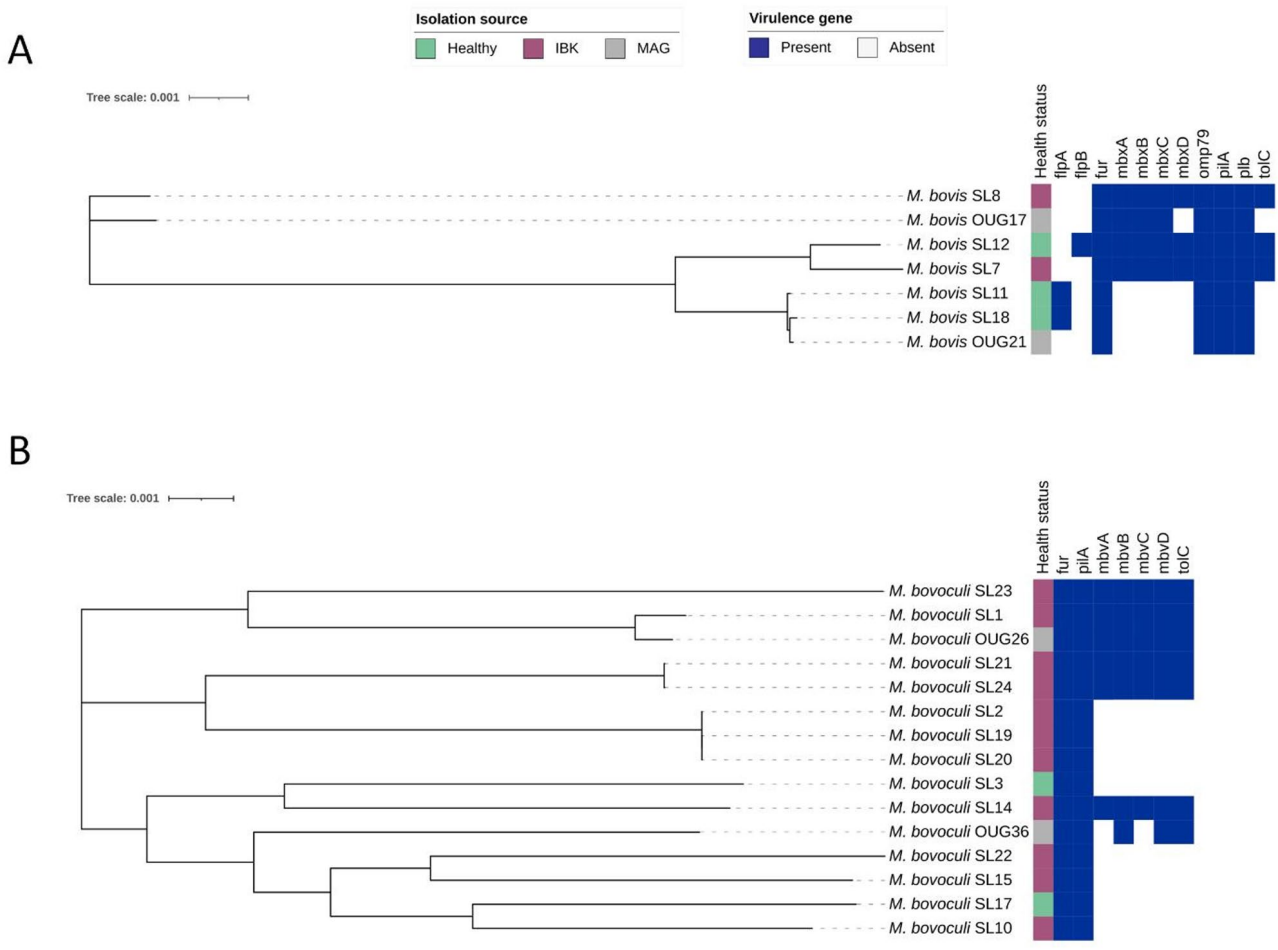
Maximum likelihood phylogenetic tree of (**A**) *Moraxella bovis* (*n* = 7) and (**B**) *Moraxella bovoculi* (*n* = 15) isolate genomes and metagenome-assembled genomes (MAG). Phylogeny was inferred from the alignment of 1,887 and 1,484 core genes for *Moraxella bovis* and *Moraxella bovoculi*, respectively. Virulence genes are also indicated as being present or absent in the isolate genomes and MAGs. The horizontal scale bar represents substitutions per nucleotide

Given their association with IBK, the core genomes of the isolates and MAGs classified as *Moraxella bovis* and *Moraxella bovoculi* were identified and used to create phylogenetic trees (Fig. 7). The core genome of the *Moraxella bovis* isolates (*n* = 5) and MAGs (*n* = 2) consisted of 1,887 genes and there were 1,484 genes in the core genome of the *Moraxella bovoculi* isolates (*n* = 13) and MAGs (*n* = 2). These genomes were also screened for the presence of 15 known virulence genes. In *Moraxella bovis*, *fur*, *omp79*, *pilA*, and *plb* were found in all genomes whereas only three isolates (SL7, SL8, and SL12) carried all four RTX-operon genes (*mbxCABD*) as well as *tolC* (Fig. 7A). One of the large *flpA* and *flpB* genes, which are typically plasmid-encoded [64], was identified in *Moraxella bovis* isolates SL11 (*flpA*), SL18 (*flpA*) and SL12 (f*lpB*). In agreement with their ANI, isolates SL11 and SL18, along with MAG OUG21, were most closely related based on the phylogenetic tree.

The *fur* and *pilA* genes were also found in all *Moraxella bovoculi* isolate genomes and MAGs (Fig. 7B). The *Moraxella bovoculi* RTX operon genes (*mbvCABD*) were identified in five isolate genomes (SL1, SL14, SL21, SL23, and SL24) and one MAG (OUG26), together with *tolC*. Consistent with their ANI values, isolates SL2, SL19, and SL20 appeared to belong to the same strain, as did SL21 and SL24.

### Inhibition of *Moraxella bovis* and *Moraxella bovoculi* growth by ocular surface commensal bacteria as determined by the agar slab method

**Fig. 8:**
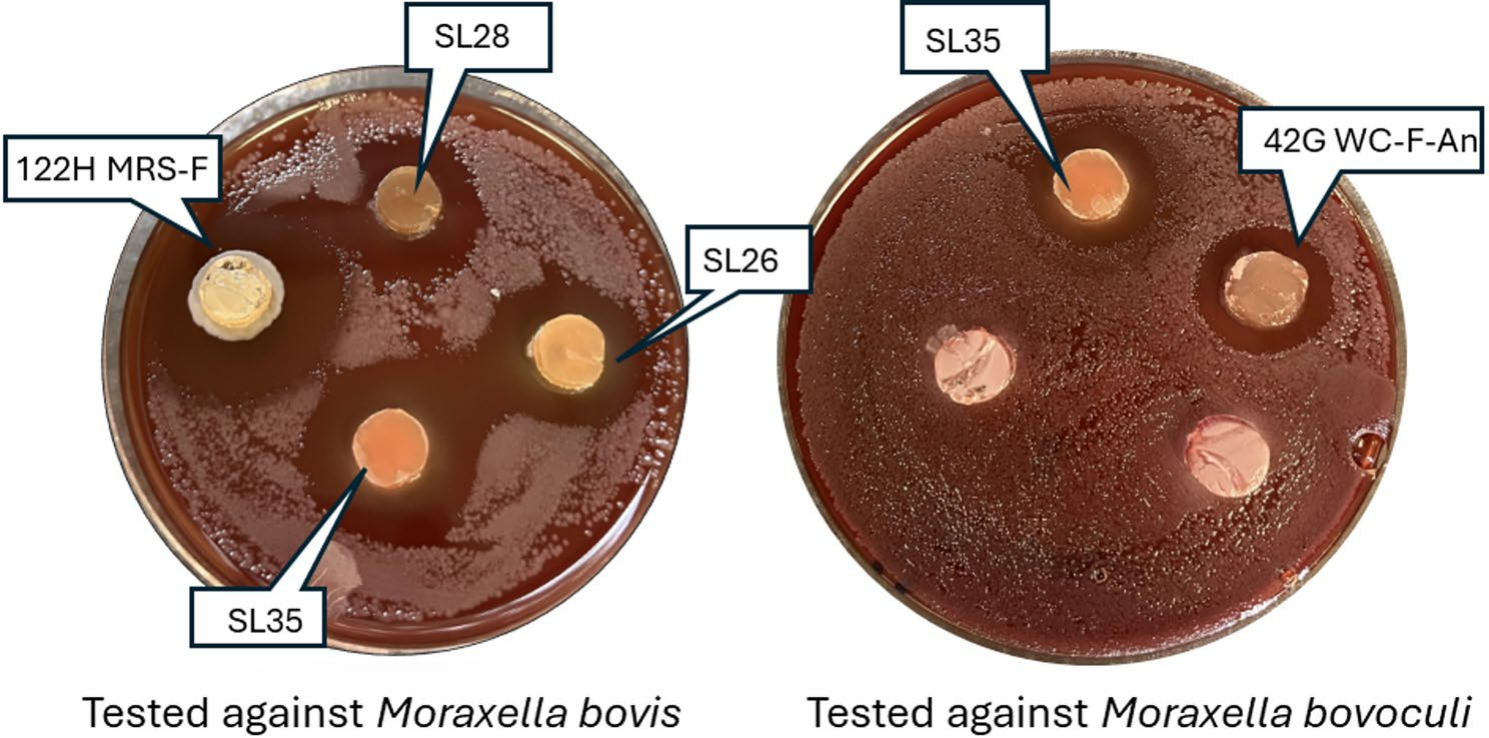
Visualization of antimicrobial activity of bovine ocular commensal bacteria against *Moraxella bovis* and *Moraxella bovoculi* using the agar slab assay. A 10-mm agar slab containing the commensal bacterial isolate was placed onto an agar plate inoculated with the target pathogen, followed by 24-h incubation. Antimicrobial activity was indicated by the presence of a zone of inhibition surrounding the agar slab. Representative commensal isolates include *Paenibacillus polymyxa* (42G WC-F-An); *Bacillus pumilus* (122H MRS-F); *Weissella paramesenteroides* (SL26); *Levilactobacillus brevis* (SL28); *Weizmannia coagulans* (SL35)

Of the 52 isolates tested for inhibition of *Moraxella* spp. using the agar slab method, 15 showed a ZOI against *Moraxella bovis*, ranging from 12.9 mm to 36.7 mm (Fig. 8; Table 3). There were also 13 isolates that inhibited the growth of *Moraxella bovoculi* with ZOIs ranging from 13 mm to 22.2 mm (Table 4). *Bacillus pumilus* (SL25 and 122 H MRS-F) and *Bacillus velezensis* (42G MRS-A Aero) exhibited the strongest inhibition against *Moraxella* spp., while *Lactiplantibacillus plantarum* (SL27), *Bacillus subtilis* (SL29), and *Weizmannia coagulans* (SL35 and SL34) showed moderate inhibition. *Lentilactobacillus buchneri* (SL36) displayed only weak inhibition.

**Table 3:** Bacterial isolates that induced zones of inhibition (ZOI) against *Moraxella bovis* using the agar slab method

Strains	Average ZOI*(mm)
*Bacillus pumilus* H MRS-F	36.7
*Bacillus sonorensis* MVC02-G	30.2
*Bacillus mojavensis* 26Z-WC-C-An	29.9
*Bacillus velezensis* 42G MRS-A-Aero	29.8
*Weissella paramesenteroides* SL16	27.7
*Lactiplantibacillus plantarum* SL27	27.4
*Levilactobacillus brevis* SL28	26.4
*Bacillus subtilis* SL29	26.0
*Limosilactobacillus fermentum* ATCC9338	22.1
*Weizmannia coagulans* SL34	21.5
*Bacillus safenis* SL31	16.7
*Desemzia incerta* 17G-WC-G-An	16.0
*Streptococcus pluranimalium* SL30	15.5
*Caldibacillus hisashii* 23D-WC-E-An	13.5
*Lentilactobacillus buchneri* SL36	12.9

*The mean ZOI was obtained from three replicates

**Table 4:** Bacterial isolates that induced zones of inhibition (ZOI) against *Moraxella bovoculi* using the agar slab method

Strain	Average ZOI* (mm)
*Bacillus pumilus* 122H MRS-F	22.2
*Bacillus velezensis* 2G-MRS-A-Ae	21.9
*Bacillus subtilis* SL29	21.1
*Lactiplantibacillus plantarum* SL27	20.6
*Limosilactobacillus fermentum* ATCC9338	19.7
*Paenibacillus polymyxa* 42G WC-F-An	19.2
*Weizmannia coagulans* SL34	18.9
*Lentilactobacillus buchneri* SL36	18.6
*Levilactobacillus brevis* SL28	16.1
*Bacillus safenis* SL31	15.5
*Weissella paramesenteroides* SL26	15.3
*Mammaliicoccus sciuri* MVC05-MRS-A-Ae	14.1
*Streptococcus pluranimalium* SL30	13.0

*The mean ZOI was obtained from three replicates

### Growth-inhibitory effects against *Moraxella bovoculi* in the presence of the culturable ocular microbiome as determined by qPCR

**Fig. 9:**
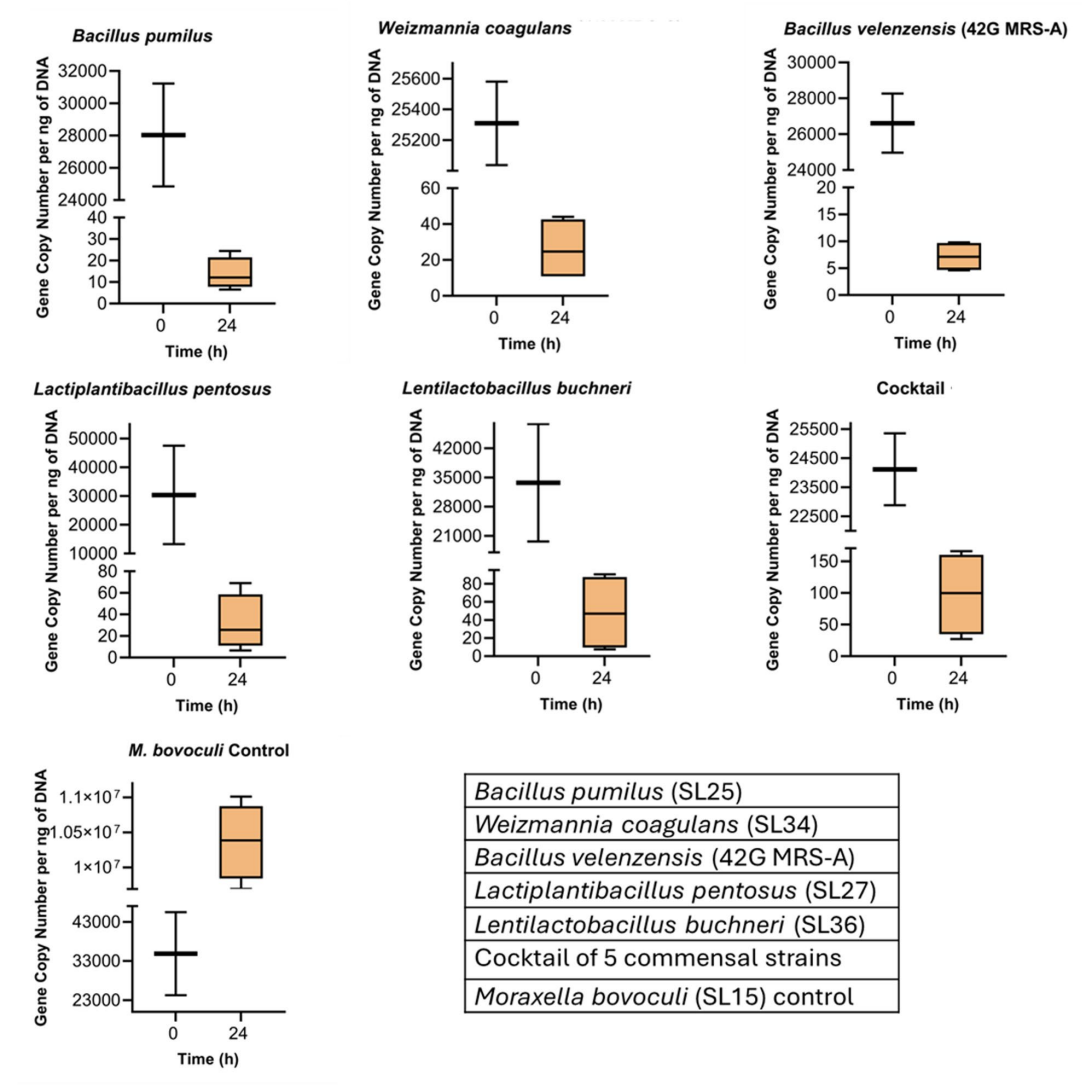
Abundance of *Moraxella* spp. as estimated by qPCR before (0 h) and after (24 h) incubation with commensal isolates and an enriched ocular bacterial community

To evaluate the potential of the candidate commensal strains to prevent proliferation of *Moraxella* spp. within a microbial community, an experiment was designed to evaluate the growth of *Moraxella bovoculi* in the presence of selected bacterial strains and the ocular microbiome. *Moraxella* abundance over a 24-h period was measured by qPCR, and the results indicated that the selected bacterial strains inhibited the growth of *Moraxella bovoculi* within an ocular microbial community (Fig. 9). Among these isolates, *B. velezensis* (42G MRS-A Aero) was most effective, reducing the concentration of *Moraxella bovoculi* by 99.97% (Fig. 9). *B. pumilus* (SL25) reduced *Moraxella bovoculi* concentration by 99.95%, *W. coagulans* (SL34) and *L. plantarum* (SL27) by 99.90%, and *L. buchneri* (SL36) by 99.86%. Additionally, a cocktail containing each of the aforementioned isolates lowered the concentration of *Moraxella* by 99.59% after 24 h of co-incubation.

## Discussion

The global cattle industry faces multifaceted challenges, including the growing issue of antimicrobial resistance in bovine bacterial pathogens. Antimicrobial resistance reduces the efficacy of antimicrobials routinely used to control infectious diseases in cattle, posing a significant threat to animal health and production and thereby emphasizing the need for alternative approaches. Advancements in high-throughput sequencing have improved our understanding of the taxonomic and functional features of the commensal microbiome across different cattle anatomical sites. As a result, the microbiome has become a key target for preventing and mitigating infectious diseases, including those caused by antimicrobial-resistant bacteria [65, 66]. To leverage the ocular microbiome for improved resistance against IBK in cattle, the first logical step is to characterize and identify its taxonomic and functional signatures in both healthy and IBK-affected cattle using culture and metagenomic sequencing methods. Next, commensal isolates from the eyes of healthy cattle should be screened for their ability to inhibit the growth of IBK-associated *Moraxella* spp. Synthetic ocular bacterial communities composed of beneficial commensal bacteria could then be developed to modulate the ocular microbiome and enhance resistance to colonization by IBK-associated pathogens.

Accordingly, in the present study, we isolated and identified a relatively large number of bacterial isolates (*n* = 351) from the ocular surface of IBK-affected and healthy beef cattle using various growth media and conditions. As such, this is the first study to characterize the culturable fraction of the bovine ocular microbiome in cattle using both aerobic and anaerobic culturing with multiple growth media. An earlier study by Gafen et al., [29] isolated bacteria from conjunctival swabs (*n* = 387) obtained from 228 IBK-affected and 159 healthy eyes in cattle (dairy and beef) using 5% sheep blood agar and incubation with 10% CO_2_ at 37 °C for 48 h. In that study, *Moraxella bovis*,* Moraxella bovoculi*,* Moraxella osloensis*,* Trueperella pyogenes*,* Bacillus* spp., and *Proteus* spp. were isolated at varying prevalences. Overall, the culturing results from Gafen and colleagues [29] and our present study suggest that the bovine ocular surface harbors a relatively diverse community of culturable bacteria.

This is further supported by culture-independent high-throughput sequencing studies. For example, a recent longitudinal study (139 days) using 16S rRNA gene sequencing of the ocular surface microbiota of 227 pre-weaned beef calves revealed the presence of a relatively rich and diverse bacterial microbiota [28]. Bacterial species within the *Moraxella*, *Mycoplasma*,* Pasteurella*, and *Acinetobacter* genera were noted to be the most relatively abundant [28]. A similar taxonomic composition of the ocular microbiota has been reported in mature and older dairy and beef cattle. Using 16S rRNA gene (V4 region) sequencing, Gafen and colleagues characterized the conjunctiva of healthy (*n* = 376) and IBK-affected cattle (*n* = 228) across different states and farms. The dominant bacterial genera on the eyes of these mature adult cattle were similar to those reported in the pre-weaned calves.

In addition, we previously characterized the ocular microbiota of neonatal beef calves and found that the ocular surface was already colonized at this age by a complex bacterial community dominated by *Streptococcus* (57.6%) [30]. Thus, results from culturing and 16S rRNA gene sequencing-based studies provide strong evidence that the bovine ocular surface is colonized by a self-sustaining, diverse, and dynamic bacterial microbiota, with *Moraxella* and *Mycoplasma* spp. being relatively abundant. The significant differences in the dominant bacterial genera identified by culturing and 16S rRNA gene sequencing highlight the necessity of using both culture-dependent (e.g., culturomics) and culture-independent methods for a comprehensive characterization of the ocular microbiome in cattle.

Although the sequencing-based studies discussed above, along with our extensive culturing, provided valuable insights into the taxonomic composition, diversity, and dynamics of the bovine ocular microbiota, 16S rRNA gene-based sequencing lacks taxonomic resolution at the species level and beyond, and does not include functional information. Therefore, in the present study, we used shotgun metagenomic sequencing to characterize the ocular surface microbiome of IBK-affected and healthy cattle. A statistically significant but modest difference was found in the microbial species composition of the two groups. This appeared to be driven by differences in the relative abundance of nine bacterial species, eight of which were enriched in the ocular microbiomes of healthy cattle. These enriched bacterial species included *B. globosum*, commonly isolated from the rumen of cattle [67] and historically used as a probiotic in pigs [68], as well as *B. licheniformis*, which has been used to improve feed digestion and milk yield in cattle [69].

Other bacterial species that were more abundant in healthy cattle included potentially pathogenic species. For example, *Lawsonella clevelandensis* has been reported to be a rare cause of abdominal, breast, spinal, and liver abscesses [70–72], and was recently reported to be an emerging cause of vascular graft infection and vascular infection in humans [71, 72]. This species has not previously been found in bovine-associated microbial communities. In addition to *L. clevelandensis* (often isolated from freshwater), *C. acnes*, a member of the human skin microbiome and considered to be both a beneficial commensal and opportunistic pathogen [73] were also enriched in the healthy cattle.

Two uncharacterized *Ruminococcus* spp. (*R*. sp900100595 and *R*. sp900316555) were also more abundant in the eyes of healthy cattle. Species within the *Ruminococcus* genus are largely host-associated and play a key role in the cattle ruminal microbiome, primarily in the breakdown of forages [74]. The only species significantly greater in abundance in the ocular microbiome of IBK-affected cattle was *A. luteus*. There is limited information regarding its colonization niche and pathogenicity; however, *A. luteus* has been reported to produce an endonuclease (AZuI), a restriction enzyme that can recognize and cleave viral DNA [75, 76]. The relationship between *A. luteus* and IBK pathogenesis therefore warrants further research. Surprisingly, the relative abundance of *Moraxella bovis* or *Moraxella bovoculi* was not significantly greater in IBK- affected cattle as compared to healthy cattle. This suggests that the presence of these species alone is not sufficient to cause IBK or that there is inter-species diversity in terms of pathogenicity.

The ocular resistome (all ARGs) did not differ between the IBK-affected and healthy cattle, although there was considerable inter-animal variability. Genes conferring resistance to the tetracyclines, macrolides, sulfonamides and aminoglycosides were most abundant in the metagenomes. Not surprisingly, these classes represent antimicrobials that are frequently used to treat cattle for various infectious diseases or conditions. For example, tetracyclines and macrolides (e.g., tulathromycin) are used to treat IBK in North America. Sulfonamides, although not used for ocular infections, are administered to treat diarrhea in nursing calves and respiratory disease in cattle [77]. Aminoglycosides are given to cattle for the treatment of various infections and are used topically in the ears and eyes and via intrauterine infusion to treat endometritis and occasionally may be infused into the udder to treat mastitis [78]. Although the ocular swabs in the present study were collected before antimicrobial treatment, our results highlight the persistence of ARGs even in the absence of direct exposure and could partially explain why certain antimicrobials (e.g., tetracycline and tulathromycin) are less effective against IBK.

The *Moraxella bovis* isolate genomes (*n* = 5) and MAGs (*n* = 2) recovered from the ocular metagenome appeared to represent unique strains based on their ANI and phylogeny, although strains SL11 and SL18 from healthy animals and MAG OUG21 were closely related. Using this same criterion, there were at least 10 strains among the *Moraxella bovoculi* isolates. This demonstrates the strain diversity of *Moraxella bovis* and *Moraxella bovoculi* on the bovine ocular surface. As such, this diversity presents a significant challenge in developing effective vaccines for IBK. The presence of different virulence factors within *Moraxella* spp. strains may help explain their association with IBK-affected or healthy cattle. Certain virulence genes, such as *fur*, *pilA*, *plb*, and *tolC*, were shared among all *Moraxella bovis* genomes. However, the presence of others including the *mbxCABD*, *flpA* and *flpB* genes varied among the five isolates and two MAGs. The *flpA* and *flpB* genes encode filamentous-haemagglutinin-like proteins that may facilitate adhesion to host cells and are typically located on a plasmid in *Moraxella bovis* [79]. While all *Moraxella bovoculi* genomes carried *fur* and *pilA*, only six carried the *mbvCABD* genes. The ubiquity of *pilA* and *tolC* in *Moraxella bovis* and *pilA* among *Moraxella bovoculi* genomes is in agreement with previous work [62]. Although the hemolysin-encoding RTX operon genes, *mbxCABD* (in *Moraxella bovis*) and *mbvCABD* (in *Moraxella bovoculi*), are associated with pathogenesis [61, 64], they were detected in *Moraxella bovis* isolates from both healthy and IBK-affected cattle.

Notably, 27% of the MAGs recovered from the ocular metagenomes were identified as *Mesomycoplasma bovoculi*, suggesting that this species is predominant in the cattle ocular microbial community. At the same time, the *Mesomycoplasma bovoculi* genome is relatively small (≈ 700 kbp) and thus less metagenomic sequencing depth is required to assemble. Although we did not isolate *Mesomycoplasma bovoculi* or any related species within the *Mesomycoplasma or Mycoplasma* genera, this is likely due to the difficulty in culturing these species in vitro [80]. *Mesomycoplasma bovoculi* (formerly *Mycoplasma bovoculi*) has been reported to predispose cattle to IBK infection [81, 82]. Therefore, characterizing the role of *Mesomycoplasma bovoculi* in ocular microbiome homeostasis, its interactions with *Moraxella bovis* and *Moraxella bovoculi*, and its role in IBK development deserve further research attention.

Human studies suggest that the ocular surface microbiome is associated with ocular health [83, 84], and that a healthy ocular microbiome may provide resistance against colonization with infectious agents [85, 86]. In this study, we identified 15 bacterial isolates that inhibited the growth of *Moraxella bovis* and 13 that inhibited *Moraxella bovoculi* growth in vitro. These isolates belonged to 10 different bacterial genera, including *Bacillus*, *Paenibacillus*,* Lactiplantibacillus*, *Streptococcus*, *Weissella*, and *Weizmannia*. *Bacillus* spp. produced the largest ZOI against *Moraxella bovis* and *Moraxella bovoculi* and members of this genus are commonly used as probiotics to inhibit pathogenic bacteria colonization through the production of antimicrobial compounds (e.g., lipopeptides and polyketides) or interference with signaling pathways [87, 88].

Direct inhibition of pathogen growth by *L. pentosus* [89], *Paenibacillus polymyxa* [90], L. *buchneri* [91], L. *brevis* [92], and *W*. *paramesenteroides* [93] has also been reported. *Weizmannia coagulans* is known for its broad-spectrum antimicrobial activity against foodborne pathogens [94–96]. Thus, the inhibition of *Moraxella bovis* and *Moraxella bovoculi* growth by these ocular bacterial species may be due to the production of antimicrobial substances such as lactic acid, bacteriocins, and hydrogen peroxide. Overall, these results suggest that the ocular surface environment is highly competitive with multiple bacteria capable of producing antimicrobial factors that can inhibit *Moraxella* spp.

Bacterial tolerance to antimicrobials can be affected by metabolic cross-feeding [97, 98]. Consequently, the antimicrobial concentrations required to inhibit or kill target bacteria differ when they are grown in the presence of other bacteria rather than in monoculture, due to the interactions within the community (e.g., competition for nutrients, biofilm formation, and production of antimicrobial substances by other bacteria) [16]. Since bacterial antimicrobial tolerance is influenced by the metabolic interdependence and interactions of different species within a microbial community, it is important to select bacterial strains that can inhibit the growth of IBK-associated pathogens in the presence of the bovine ocular microbiome. Here, we selected five isolates (*B. pumilus*, *B. velezensis*, *L. plantarum*, *L. buchneri* and *W. coagulans*) that displayed relatively strong inhibition of *Moraxella bovis* and *Moraxella bovoculi*. These isolates were then further evaluated for their ability to inhibit the growth of *Moraxella bovoculi* in the presence of the bovine ocular microbiome. The growth of *Moraxella bovoculi* co-cultured with the ocular microbiome was inhibited by all five strains, as well as a mixture of these strains. This suggests that the selected isolates have the potential to inhibit *Moraxella* spp. on the ocular surface.

Despite these findings, several limitations should be acknowledged. First, all samples were collected from cattle in North Dakota, which may limit the generalizability of the results to other geographic regions, management systems, or environmental conditions. Second, this study was cross-sectional in design, capturing a single time point rather than longitudinal dynamics; therefore, temporal changes in the ocular microbiome and causal relationships with IBK development cannot be fully assessed. Third, although shotgun metagenomic sequencing provided functional and species-level resolution, a high proportion of host DNA (92.9–96.4%) reduced sequencing depth for microbial reads, potentially limiting the detection of low-abundance taxa and contributing to the incomplete recovery of some cultured isolates as metagenome-assembled genomes. In addition, the imbalance in sample size between IBK-affected (*n* = 37) and healthy (*n* = 12) cattle in the metagenomic subset may have reduced statistical power to detect differences in microbial composition and function. Finally, the associations observed between specific bacterial taxa and ocular health status are correlative, and causal relationships cannot be inferred. Future studies incorporating longitudinal designs, improved host DNA depletion strategies, balanced sampling, and in vivo validation will be important to confirm and extend these findings.

## Conclusion

In summary, we characterized the ocular surface microbiome of IBK-affected and healthy cattle using culture-dependent and culture-independent methods. Eight bacterial species were relatively more abundant in the ocular microbiome of healthy cattle based on metagenomic sequencing, including *B. globosum*,* B. licheniformis*,* Ruminococcus* sp900316555, and *Ruminococcus* sp900100595. Using large-scale aerobic and anaerobic culturing, we identified 351 isolates representing 61 different genera. Of the 52 bacterial isolates screened for inhibition of *Moraxella bovoculi* and *Moraxella bovis*, 17 inhibited the growth of one of these species. Overall, our results provide deeper insight into the ocular microbiome in cattle. This knowledge is important for the development of antimicrobial alternatives for preventing and treating IBK in cattle.

## Electronic Supplementary Material

Below is the link to the electronic supplementary material.

Supplementary Material 1

## Data Availability

Raw sequence data and genome assemblies are available on NCBI under BioProject accession PRJNA1128155. Other data supporting the findings of this study are presented within the paper and in the supplementary information files.
