# Supplementary material for: The bovine ocular microbiome: a multi-approach study of composition and antimicrobial activity
Source: Anim Microbiome. 2026 Jun 15;8:85. doi: 10.1186/s42523-026-00587-0 (PMC13330061; doi:10.1186/s42523-026-00587-0)
Supplement: Supplementary file 1 — Supplementary Material 1 [file 42523_2026_587_MOESM1_ESM.docx]

**Supplementary Table S1.** Summary of metagenome-assembled genomes (MAGs) recovered from the metagenomes of swabs of the bovine ocular surface.

| **MAG name** | **Species** | **Antimicrobial resistance genes** | **Healthy metagenomes (% relative abundance)** | **IBK-affected metagenomes (% relative abundance)** | **No. contigs** | **Largest contig (bp)** | **Total length (bp)** | **Completeness (%)** | **Contamination (%)** |
| --- | --- | --- | --- | --- | --- | --- | --- | --- | --- |
| OUG1 | d__Bacteria;p__Pseudomonadota;c__Gammaproteobacteria;o__Cardiobacteriales;f__;g__;s__ |  | 18.971 ± 8.901 | 0.571 ± 0.567 | 23 | 208,281 | 1,009,203 | 99.4 | 0.23 |
| OUG2 | d__Bacteria;p__Bacillota_A;c__Clostridia;o__Tissierellales;f__Peptoniphilaceae;g__Helcococcus;s__ |  | 0.004 ± 0.003 | 0.304 ± 0.135 | 61 | 135,850 | 1,456,752 | 100 | 1.6 |
| OUG3 | d__Bacteria;p__Bacillota;c__Bacilli;o__Lactobacillales;f__Streptococcaceae;g__Lactococcus;s__*Lactococcus lactis* | lmr(D) | ND | 0.17 ± 0.096 | 112 | 135,752 | 2,368,110 | 100 | 0.16 |
| OUG4 | d__Bacteria;p__Bacillota_I;c__Bacilli_A;o__Mycoplasmatales;f__Metamycoplasmataceae;g__Mycoplasmopsis;s__*Mycoplasmopsis bovis* |  | 0.003 ± 0.003 | 1.214 ± 1.166 | 43 | 65,714 | 861,575 | 97.15 | 0.54 |
| OUG5 | d__Bacteria;p__Bacillota;c__Bacilli;o__Lactobacillales;f__Aerococcaceae;g__Globicatella;s__ |  | 0.003 ± 0.002 | 0.058 ± 0.023 | 203 | 55,936 | 2,082,830 | 97.17 | 0.17 |
| OUG6 | d__Bacteria;p__Bacillota;c__Bacilli;o__Lactobacillales;f__Streptococcaceae;g__Streptococcus;s__*Streptococcus ruminantium* | patA, patB | 0.001 ± 0.001 | 0.172 ± 0.104 | 90 | 88,153 | 1,843,796 | 99.72 | 0.06 |
| OUG7 | d__Bacteria;p__Bacillota;c__Bacilli;o__Lactobacillales;f__Carnobacteriaceae;g__Desemzia;s__Desemzia incerta | lnu(C), norC | 0.019 ± 0.007 | 0.038 ± 0.014 | 301 | 47,920 | 2,093,804 | 94.03 | 3.73 |
| OUG8 | d__Bacteria;p__Bacillota_I;c__Bacilli_A;o__Mycoplasmatales;f__Metamycoplasmataceae;g__Mesomycoplasma;s__*Mesomycoplasma bovoculi* |  | 0.398 ± 0.227 | 1.072 ± 0.286 | 118 | 14,491 | 496,564 | 90.52 | 3.75 |
| OUG9 | d__Bacteria;p__Pseudomonadota;c__Gammaproteobacteria;o__Enterobacterales;f__Pasteurellaceae;g__;s__ |  | 3.618 ± 2.379 | 1.765 ± 1.033 | 18 | 281,223 | 1,020,278 | 100 | 0 |
| OUG10 | d__Bacteria;p__Actinomycetota;c__Actinomycetes;o__Propionibacteriales;f__Propionibacteriaceae;g__Cutibacterium;s__*Cutibacterium acnes* |  | 0.32 ± 0.079 | 0.128 ± 0.054 | 294 | 41,136 | 2,367,111 | 96.08 | 2.63 |
| OUG11 | d__Bacteria;p__Pseudomonadota;c__Gammaproteobacteria;o__Enterobacterales;f__Pasteurellaceae;g__Mannheimia;s__*Mannheimia pernigra* | qacG | 0.217 ± 0.11 | 0.717 ± 0.197 | 77 | 125,774 | 2,011,479 | 99.99 | 0.04 |
| OUG12 | d__Bacteria;p__Pseudomonadota;c__Gammaproteobacteria;o__Xanthomonadales;f__Xanthomonadaceae;g__Luteimonas_D;s__Luteimonas_D excrementigallinarum | qacG, qacJ | 0.009 ± 0.004 | 0.143 ± 0.128 | 407 | 33,161 | 2,533,838 | 90.97 | 3.42 |
| OUG13 | d__Bacteria;p__Bacillota_I;c__Bacilli_A;o__Mycoplasmatales;f__Metamycoplasmataceae;g__Mesomycoplasma;s__*Mesomycoplasma bovoculi* |  | 0.526 ± 0.265 | 1.685 ± 0.593 | 4 | 548,266 | 753,345 | 99.66 | 0.06 |
| OUG14 | d__Bacteria;p__Bacillota;c__Bacilli;o__Lactobacillales;f__Aerococcaceae;g__WM01;s__ |  | 0.011 ± 0.005 | 0.092 ± 0.042 | 70 | 138,049 | 1,406,075 | 96.22 | 0.02 |
| OUG15 | d__Bacteria;p__Actinomycetota;c__Actinomycetes;o__Propionibacteriales;f__Propionibacteriaceae;g__Cutibacterium;s__*Cutibacterium acnes* |  | 0.236 ± 0.059 | 0.134 ± 0.06 | 347 | 28,371 | 2,265,735 | 91.72 | 2.25 |
| OUG16 | d__Bacteria;p__Bacillota_A;c__Clostridia;o__Tissierellales;f__Peptoniphilaceae;g__Helcococcus;s__ |  | 0.002 ± 0.002 | 0.121 ± 0.066 | 56 | 76,267 | 1,050,207 | 95.19 | 2.2 |
| OUG17 | d__Bacteria;p__Pseudomonadota;c__Gammaproteobacteria;o__Pseudomonadales;f__Moraxellaceae;g__Moraxella;s__*Moraxella bovis* | blaBRO-1, qacG, rsmA | 0.17 ± 0.064 | 1.156 ± 0.35 | 220 | 131,128 | 2,335,751 | 95.98 | 0.02 |
| OUG18 | d__Bacteria;p__Pseudomonadota;c__Gammaproteobacteria;o__Cardiobacteriales;f__Cardiobacteriaceae;g__;s__ |  | 0.069 ± 0.037 | 2.082 ± 1.36 | 26 | 236,744 | 1,664,769 | 100 | 0.04 |
| OUG19 | d__Bacteria;p__Pseudomonadota;c__Gammaproteobacteria;o__Cardiobacteriales;f__Cardiobacteriaceae;g__Suttonella;s__ |  | 0.434 ± 0.399 | 1.084 ± 0.566 | 119 | 65,203 | 2,097,414 | 99.99 | 0.01 |
| OUG20 | d__Bacteria;p__Bacillota_I;c__Bacilli_A;o__Mycoplasmatales;f__Metamycoplasmataceae;g__Mesomycoplasma;s__*Mesomycoplasma bovoculi* |  | 0.786 ± 0.245 | 1.349 ± 0.44 | 47 | 86,767 | 641,313 | 94.57 | 0.16 |
| OUG21 | d__Bacteria;p__Pseudomonadota;c__Gammaproteobacteria;o__Pseudomonadales;f__Moraxellaceae;g__Moraxella;s__*Moraxella bovis* | blaBRO-1, qacG, rsmA | 0.324 ± 0.18 | 1.206 ± 0.315 | 180 | 77,909 | 2,515,507 | 99.98 | 0.12 |
| OUG22 | d__Bacteria;p__Bacillota_I;c__Bacilli_A;o__Mycoplasmatales;f__Metamycoplasmataceae;g__Mesomycoplasma;s__Mesomycoplasma bovoculi |  | 0.758 ± 0.353 | 0.804 ± 0.173 | 58 | 36,339 | 609,064 | 92.01 | 0.02 |
| OUG23 | d__Bacteria;p__Bacillota_I;c__Bacilli_A;o__Mycoplasmatales;f__Metamycoplasmataceae;g__Mesomycoplasma;s__Mesomycoplasma bovoculi |  | 0.455 ± 0.223 | 0.958 ± 0.234 | 89 | 38,343 | 672,390 | 94.39 | 3.02 |
| OUG24 | d__Bacteria;p__Bacillota_I;c__Bacilli_A;o__Mycoplasmatales;f__Mycoplasmoidaceae;g__Malacoplasma;s__ |  | 0.405 ± 0.234 | 1.967 ± 1.043 | 21 | 270,407 | 833,992 | 95.12 | 0.01 |
| OUG25 | d__Bacteria;p__Pseudomonadota;c__Gammaproteobacteria;o__Burkholderiales;f__Neisseriaceae;g__;s__ | qacG | 0.006 ± 0.006 | 0.337 ± 0.325 | 113 | 79,710 | 2,105,832 | 99.52 | 0.03 |
| OUG26 | d__Bacteria;p__Pseudomonadota;c__Gammaproteobacteria;o__Pseudomonadales;f__Moraxellaceae;g__Moraxella;s__Moraxella bovoculi | blaBRO-1, qacG | 0.133 ± 0.053 | 2.69 ± 0.788 | 110 | 84,244 | 1,982,950 | 99.97 | 0.73 |
| OUG27 | d__Bacteria;p__Bacillota_I;c__Bacilli_A;o__Mycoplasmatales;f__Metamycoplasmataceae;g__Mesomycoplasma;s__Mesomycoplasma bovoculi |  | 1.079 ± 0.537 | 0.907 ± 0.208 | 35 | 72,377 | 720,292 | 96.55 | 0.03 |
| OUG28 | d__Bacteria;p__Pseudomonadota;c__Gammaproteobacteria;o__Enterobacterales;f__Pasteurellaceae;g__Mannheimia;s__Mannheimia sp000521605 | qacG | ND | 0.144 ± 0.133 | 153 | 66,003 | 2,169,942 | 99.62 | 0.04 |
| OUG29 | d__Bacteria;p__Bacillota_I;c__Bacilli_A;o__Mycoplasmatales;f__Metamycoplasmataceae;g__Mesomycoplasma;s__Mesomycoplasma bovoculi |  | 0.493 ± 0.252 | 1.85 ± 1.015 | 3 | 578,471 | 752,162 | 99.45 | 0.06 |
| OUG30 | d__Bacteria;p__Bacillota_I;c__Bacilli_A;o__Mycoplasmatales;f__Metamycoplasmataceae;g__Mesomycoplasma;s__Mesomycoplasma bovoculi |  | 1.053 ± 0.595 | 0.75 ± 0.161 | 42 | 96,286 | 669,555 | 96.76 | 0.03 |
| OUG31 | d__Bacteria;p__Bacillota_I;c__Bacilli_A;o__Mycoplasmatales;f__Metamycoplasmataceae;g__Mesomycoplasma;s__ |  | 0.65 ± 0.594 | 0.061 ± 0.016 | 10 | 193,948 | 726,380 | 96.74 | 0.02 |
| OUG32 | d__Bacteria;p__Bacillota_I;c__Bacilli_A;o__Mycoplasmatales;f__Metamycoplasmataceae;g__Mesomycoplasma;s__Mesomycoplasma bovoculi |  | 0.5 ± 0.258 | 0.966 ± 0.211 | 68 | 53,179 | 685,976 | 98.03 | 0.93 |
| OUG33 | d__Bacteria;p__Bacillota;c__Bacilli;o__Bacillales_D;f__Amphibacillaceae;g__Lentibacillus_C;s__Lentibacillus_C daqui | ant(9)-Ia, blaIII, erm(A), fexA, msr(I), vanG | ND | 0.692 ± 0.692 | 96 | 287,000 | 3,613,880 | 100 | 0.27 |
| OUG34 | d__Bacteria;p__Bacteroidota;c__Bacteroidia;o__Flavobacteriales;f__Weeksellaceae;g__JABCPE02;s__ |  | 0.037 ± 0.029 | 0.18 ± 0.053 | 257 | 30,474 | 1,714,935 | 92.19 | 3.38 |
| OUG35 | d__Bacteria;p__Bacteroidota;c__Bacteroidia;o__Flavobacteriales;f__Flavobacteriaceae;g__Capnocytophaga;s__ | mphE | 0.012 ± 0.011 | 0.162 ± 0.076 | 243 | 64,400 | 2,503,417 | 98.19 | 1.37 |
| OUG36 | d__Bacteria;p__Pseudomonadota;c__Gammaproteobacteria;o__Pseudomonadales;f__Moraxellaceae;g__Moraxella;s__Moraxella bovoculi | floR, qacG | 0.072 ± 0.038 | 3.08 ± 1.021 | 92 | 182,673 | 1,927,070 | 93.5 | 0.39 |
| OUG37 | d__Bacteria;p__Bacillota_I;c__Bacilli_A;o__Mycoplasmatales;f__Metamycoplasmataceae;g__Mesomycoplasma;s__Mesomycoplasma bovoculi |  | 1.8 ± 1.502 | 2.112 ± 0.883 | 1 | 756,973 | 756,973 | 99.33 | 0.06 |

The relative abundance (%) of the MAGs in the ocular surface metagenomes of healthy (n = 12) and infectious bovine keratoconjunctivitis (IBK; n = 37) is also included. ND = not detected.

**Supplementary Table S2.** The whole-genome sequencing summary and basic properties of bacterial isolates (n = 31) recovered from swabs of the bovine ocular surface.

| Isolate ID | Species | Strain | Healthy metagenomes (% relative abundance) | IBK-affected metagenomes (% relative abundance) | Antimicrobial resistance genes | Plasmids | No. contigs | Total length (bp) | N50 (bp) | GC (%) | Completeness (%) | Contamination (%) |
| --- | --- | --- | --- | --- | --- | --- | --- | --- | --- | --- | --- | --- |
| SL16 | *Weissella paramesenteroides* | C | ND | ND |  |  | 29 | 1,895,277 | 108,017 | 37.99 | 100 | 0.21 |
| SL25 | *Bacillus pumilus* |  | ND | 0.006 ± 0.004 | *blaBPU-1, cat86, qacG, qacJ* | **Plasmid AG691** - 29,242 bp; non-mobilizable; CP022320.2 **Plasmid (novel)** - 6,723 bp; relaxase type: MOBV; mobilizable; rep type: Inc13; MF503688.1 **Plasmid AC057** - 6,448 bp; relaxase type: MOBV; rep type: Inc13; NC_004932.1 **Plasmid AE387** - 1,565 bp; non-mobilizable; CP042065.1 | 71 | 4,101,060 | 123,106 | 41.01 | 100 | 0.35 |
| SL26 | *Weissella paramesenteroides* | C | ND | ND |  |  | 29 | 1,895,019 | 115,722 | 37.99 | 100 | 0.21 |
| SL28 | *Levilactobacillus brevis* |  | ND | ND | *nimA* |  | 29 | 2,358,688 | 154,024 | 45.96 | 99.98 | 0.21 |
| SL29 | *Bacillus subtilis* |  | ND | 0.002 ± 0.002 | *aadK, blaI, blt, bmr, fosBx1, lmr(B), mphK, mprF, qacG, qacJ, tet(45), tmrB, vmlR, ykkC, ykkD* | **Plasmid AE387** - 1,565 bp; non-mobilizable; CP042065.1 | 11 | 4,210,211 | 1,043,711 | 43.37 | 100 | 0.07 |
| SL11 | *Moraxella bovis* |  | 0.359 ± 0.182 | 0.545 ± 0.109 | *blaBRO-1, qacG* | **Plasmid AC803** - 35,674 bp; relaxase type: MOBP; mobilizable; CP030242.1 **Plasmid AB842** - 4,735 bp; relaxase type: MOBQ; mobilizable; GQ998872.1 | 132 | 2,919,065 | 42,583 | 43.63 | 100 | 0.04 |
| SL18 | *Moraxella bovis* |  | 0.318 ± 0.186 | 0.462 ± 0.103 | *blaBRO-1, qacG, rsmA* | **Plasmid AC803** - 35,674 bp; relaxase type: MOBP; mobilizable; CP030242.1 **Plasmid AB842** - 4,735 bp; relaxase type: MOBQ; mobilizable; GQ998872.1 | 131 | 2,911,751 | 45,412 | 43.62 | 100 | 0.12 |
| SL12 | *Moraxella bovis* |  | 0.203 ± 0.109 | 0.778 ± 0.182 | *blaBRO-1, qacG* | **Plasmid AC803** - 32,265 bp; relaxase type: MOBP; mobilizable; CP030242.1 | 106 | 2,702,156 | 41,298 | 43.89 | 100 | 0.07 |
| SL8 | *Moraxella bovis* |  | 0.193 ± 0.076 | 0.857 ± 0.329 | *blaBRO-1, qacG, rsmA* |  | 90 | 2,497,040 | 57,887 | 44.09 | 99.99 | 0 |
| SL7 | *Moraxella bovis* |  | 0.189 ± 0.099 | 0.649 ± 0.147 | *qacG, rsmA* |  | 118 | 2,785,717 | 48,954 | 43.83 | 99.99 | 0.08 |
| SL1 | *Moraxella bovoculi* |  | 0.048 ± 0.025 | 0.782 ± 0.242 | *blaBRO-1, qacG* | **Plasmid AH517** - 2,915 bp; predicted to be non-mobilizable; CP031768.1 | 25 | 2,104,208 | 499,673 | 45.59 | 100 | 0.05 |
| SL23 | *Moraxella bovoculi* |  | 0.035 ± 0.021 | 0.643 ± 0.216 | *qacG* |  | 34 | 2,032,532 | 159,579 | 45.7 | 100 | 0.03 |
| SL14 | *Moraxella bovoculi* |  | 0.031 ± 0.015 | 0.572 ± 0.158 | *qacG* | **Plasmid AH166** - 47,786 bp; relaxase type: MOBF; mobilizable; CP011375.1 | 30 | 2,080,510 | 146,116 | 45.57 | 100 | 0.04 |
| SL21 | *Moraxella bovoculi* | B | 0.03 ± 0.016 | 0.709 ± 0.341 | *qacG, tet(45)* |  | 29 | 2,111,526 | 156,115 | 45.55 | 100 | 0.04 |
| SL17 | *Moraxella bovoculi* |  | 0.03 ± 0.012 | 0.572 ± 0.161 | *qacG* |  | 17 | 2,013,725 | 258,537 | 45.73 | 100 | 0.03 |
| SL3 | *Moraxella bovoculi* |  | 0.028 ± 0.015 | 0.523 ± 0.149 | *blaBRO-1, qacG* |  | 24 | 2,030,971 | 177,562 | 45.58 | 100 | 0.04 |
| SL24 | *Moraxella bovoculi* | B | 0.027 ± 0.015 | 0.697 ± 0.335 | *qacG, tet(45)* |  | 27 | 2,096,145 | 156,127 | 45.54 | 100 | 0.04 |
| SL15 | *Moraxella bovoculi* |  | 0.026 ± 0.012 | 0.588 ± 0.172 | *qacG* |  | 27 | 2,061,821 | 207,507 | 45.63 | 100 | 0.06 |
| SL10 | *Moraxella bovoculi* |  | 0.025 ± 0.012 | 0.551 ± 0.159 | *qacG* |  | 15 | 2,027,445 | 269,129 | 45.7 | 100 | 0.02 |
| SL22 | *Moraxella bovoculi* |  | 0.024 ± 0.012 | 0.592 ± 0.168 | *qacG* |  | 30 | 2,087,268 | 206,171 | 45.57 | 100 | 0.01 |
| SL19 | *Moraxella bovoculi* | A | 0.017 ± 0.009 | 0.345 ± 0.099 | *qacG* |  | 23 | 2,033,910 | 183,280 | 45.6 | 100 | 0.06 |
| SL2 | *Moraxella bovoculi* | A | 0.016 ± 0.009 | 0.344 ± 0.099 |  |  | 26 | 2,033,464 | 154,732 | 45.61 | 100 | 0.06 |
| SL20 | *Moraxella bovoculi* | A | 0.016 ± 0.009 | 0.344 ± 0.099 | *qacG* |  | 23 | 2,033,978 | 181,698 | 45.6 | 100 | 0.06 |
| SL33 | *Bacillus licheniformis* |  | 0.011 ± 0.005 | 0.004 ± 0.002 | *blaIII, fosBx1, qacG* |  | 26 | 4,290,250 | 490,787 | 45.94 | 100 | 0.21 |
| SL32 | *Bacillus licheniformis* |  | 0.01 ± 0.005 | 0.004 ± 0.002 | *blaIII, erm(D), fosBx1, qacG* |  | 30 | 4,151,661 | 310,670 | 46.14 | 100 | 0.18 |
| SL35 | *Weizmannia coagulans* |  | 0.008 ± 0.006 | 0.005 ± 0.005 |  |  | 104 | 3,376,215 | 59,233 | 46.48 | 100 | 0.35 |
| SL34 | *Weizmannia coagulans* |  | 0.007 ± 0.006 | 0.005 ± 0.005 |  |  | 101 | 3,407,310 | 55,217 | 46.4 | 100 | 0.12 |
| SL27 | *Lactiplantibacillus plantarum* |  | 0.004 ± 0.003 | ND |  |  | 31 | 3,254,160 | 317,841 | 44.48 | 100 | 0.63 |
| SL30 | *Streptococcus pluranimalium* | | 0.003 ± 0.003 | 0.037 ± 0.011 |  |  | 18 | 1,917,894 | 1,012,382 | 38.53 | 99.96 | 0.25 |
| SL36 | *Lentilactobacillus buchneri* |  | 0.003 ± 0.003 | 0.049 ± 0.034 |  | **Plasmid AH518** - 46,348 bp; non-mobilizable; CP043613.1  **Plasmid (novel)** - 43,644 bp; rep type: rep_cluster_707; conjugative; relaxase type: MOBQ; CP029967 **Plasmid AB201 -** 5,342 bp; non-mobilizable; CP035273.1 | 53 | 2,480,928 | 245,829 | 44.07 | 100 | 0.46 |
| SL31 | *Bacillus safensis* |  | 0.001 ± 0.001 | ND | *cat86, qacG, qacJ* |  | 32 | 3,682,248 | 209,466 | 41.5 | 100 | 0.41 |

Uppercase letters indicate isolates from the same strain (≥ 99.99% average nucleotide identity). The relative abundance (%) of the isolate genomes in the ocular surface metagenomes of healthy (n = 12) and infectious bovine keratoconjunctivitis (IBK; n = 37) is also included. ND = not detected.

**
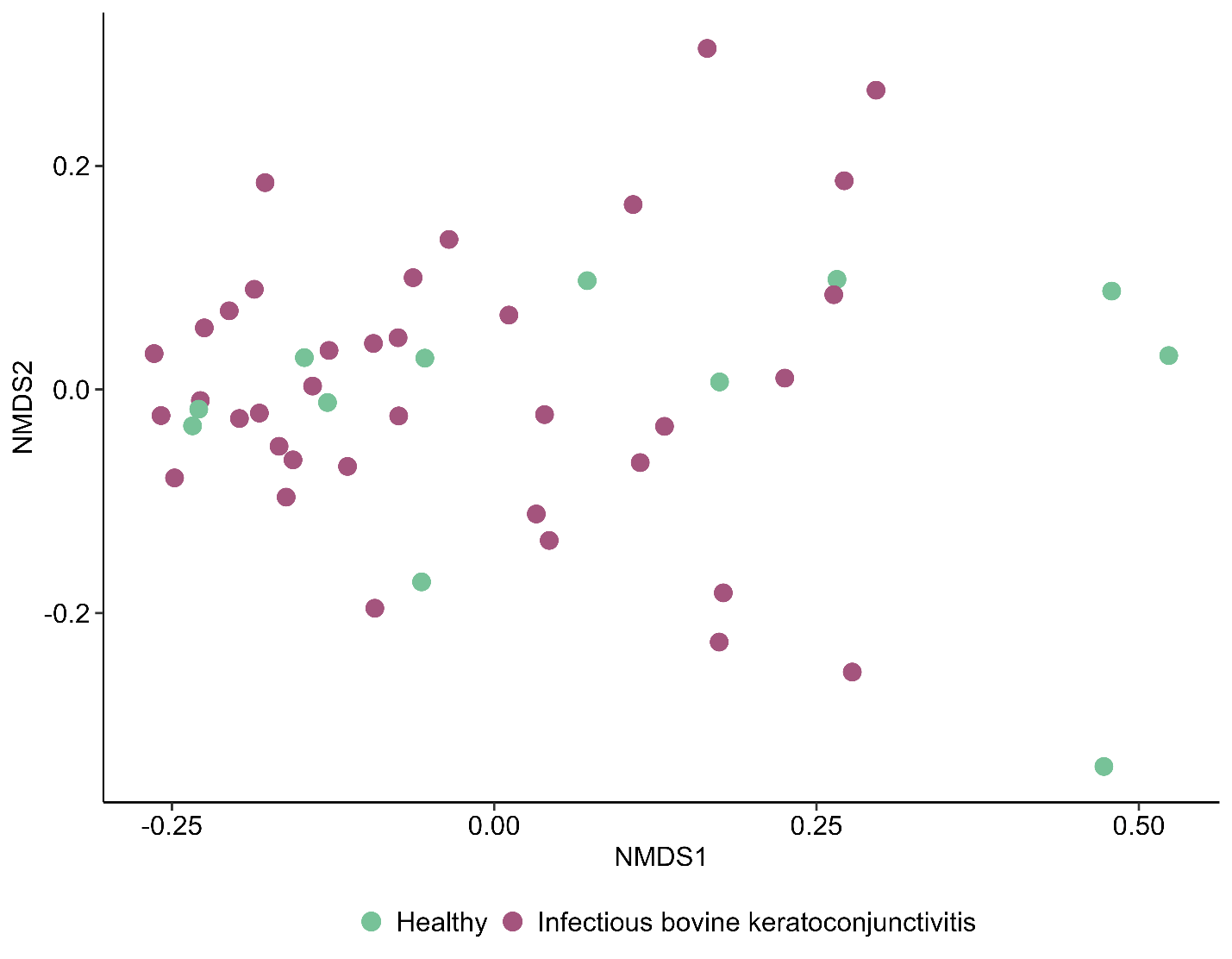
**

**Supplementary Figure S1.** Non-metric multidimensional scaling (NMDS) plot of Bray Curtis dissimilarity calculated from KEGG pathway abundances (copies per million reads) in the ocular surface microbiomes of infectious bovine keratoconjunctivitis-affected vs. healthy cattle.
